# Rapid whole-heart CMR with single volume super-resolution

**DOI:** 10.1186/s12968-020-00651-x

**Published:** 2020-08-03

**Authors:** Jennifer A. Steeden, Michael Quail, Alexander Gotschy, Kristian H. Mortensen, Andreas Hauptmann, Simon Arridge, Rodney Jones, Vivek Muthurangu

**Affiliations:** 1 UCL Centre for Cardiovascular Imaging, Institute of Cardiovascular Science, University College London, 30 Guildford Street, London, WC1N 1EH UK; 2 Great Ormond Street Hospital, London, WC1N 3JH UK; 3 Institute for Biomedical Engineering, University and ETH Zurich, Zurich, Switzerland; 4 Department of Computer Science, University College London, London, WC1E 6BT UK; 5 Research Unit of Mathematical Sciences, University of Oulu, Oulu, Finland

**Keywords:** Super-resolution, Whole-heart imaging, Machine learning, Rapid imaging, Convolutional neural network

## Abstract

**Background:**

Three-dimensional, whole heart, balanced steady state free precession (WH-bSSFP) sequences provide delineation of intra-cardiac and vascular anatomy. However, they have long acquisition times. Here, we propose significant speed-ups using a deep-learning single volume super-resolution reconstruction, to recover high-resolution features from rapidly acquired low-resolution WH-bSSFP images.

**Methods:**

A 3D residual U-Net was trained using synthetic data, created from a library of 500 high-resolution WH-bSSFP images by simulating 50% slice resolution and 50% phase resolution. The trained network was validated with 25 synthetic test data sets. Additionally, prospective low-resolution data and high-resolution data were acquired in 40 patients. In the prospective data, vessel diameters, quantitative and qualitative image quality, and diagnostic scoring was compared between the low-resolution, super-resolution and reference high-resolution WH-bSSFP data.

**Results:**

The synthetic test data showed a significant increase in image quality of the low-resolution images after super-resolution reconstruction. Prospectively acquired low-resolution data was acquired ~× 3 faster than the prospective high-resolution data (173 s vs 488 s). Super-resolution reconstruction of the low-resolution data took < 1 s per volume. Qualitative image scores showed super-resolved images had better edge sharpness, fewer residual artefacts and less image distortion than low-resolution images, with similar scores to high-resolution data. Quantitative image scores showed super-resolved images had significantly better edge sharpness than low-resolution or high-resolution images, with significantly better signal-to-noise ratio than high-resolution data. Vessel diameters measurements showed over-estimation in the low-resolution measurements, compared to the high-resolution data. No significant differences and no bias was found in the super-resolution measurements in any of the great vessels. However, a small but significant for the underestimation was found in the proximal left coronary artery diameter measurement from super-resolution data. Diagnostic scoring showed that although super-resolution did not improve accuracy of diagnosis, it did improve diagnostic confidence compared to low-resolution imaging.

**Conclusion:**

This paper demonstrates the potential of using a residual U-Net for super-resolution reconstruction of rapidly acquired low-resolution whole heart bSSFP data within a clinical setting. We were able to train the network using synthetic training data from retrospective high-resolution whole heart data. The resulting network can be applied very quickly, making these techniques particularly appealing within busy clinical workflow. Thus, we believe that this technique may help speed up whole heart CMR in clinical practice.

## Background

Three-dimensional whole heart, balanced steady state free precession (WH-bSSFP) imaging is an important part of the cardiovascular magnetic resonance (CMR) imaging protocol in congenital heart disease [1]. This is because WH-bSSFP provides excellent delineation of both intra-cardiac and vascular anatomy. However, WH-bSSFP sequences are usually cardiac triggered and respiratory navigated, resulting in long acquisition times (up to 10 min).

Significant speed-ups can be achieved through the use of non-Cartesian sampling (i.e. spiral [2] or radial [3]) or data under-sampling with state-of-the-art reconstruction strategies (i.e. compressed sensing [4]). Unfortunately, these methods require major sequence modifications and are often handicapped by long reconstruction times, even with the use of modern computing (i.e. graphics processing units [5]). An alternative approach is single volume super-resolution reconstruction (SRR), where high-resolution features are recovered from rapidly acquired low-resolution data. The benefits of SRR is that it can be performed as a simple post-processing step without any sequence modification. However, conventional algorithms often produce unrealistic looking images, limiting the utility of this method [6]. Recently, machine learning has transformed SRR with the ability to produce realistic high-resolution images from low-resolution data [7–9].

In this study, we use a deep-learning SRR approach to reconstruct high-resolution data from rapidly acquired low-resolution WH-bSSFP images. This was achieved by first creating a ‘synthetic’ low-resolution training data set from a library of reference standard high-resolution WH-bSSFP images. The paired data were then used to train a convolutional neural network (CNN) to map between low-resolution and high-resolution images (super-resolution). The aims of this study were to: i) Assess the accuracy of deep learning single volume SRR for recovering high-resolution data from synthetically down-sampled WH-bSSFP data, ii) Assess the robustness of the resultant network, at recovering high-resolution data from different resolution input data, iii) Assess the feasibility of using deep learning single volume SRR for reconstruction of prospectively acquired low-resolution WH-bSSFP data, and iv) Compare acquisition time, image quality, accuracy of vessel diameter measurements and diagnostic value from single volume SRR, compared to low-resolution and reference standard high-resolution WH-bSSFP images.

## Methods

### Network architecture

**Fig. 1:**
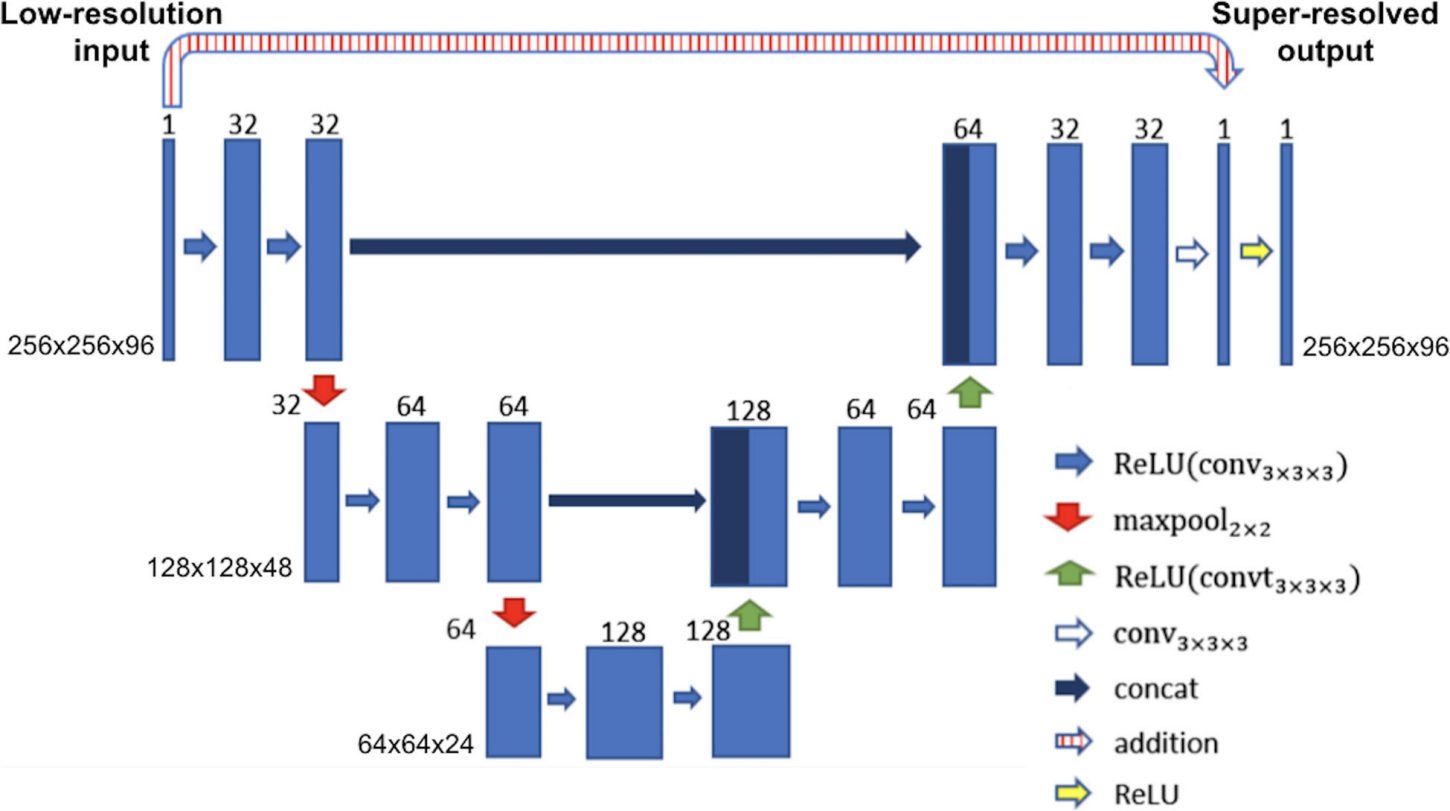
Network architecture. Chosen residual U-Net architecture used for 3D single volume super-resolution. The input is given by the low-resolution WH-bSSFP images. The numbers on top of the blue bars denote the number of channels for each layer. The resolution for each multilevel decomposition is shown on the left. Each convolutional layer is equipped with a Rectified Linear Unit as nonlinearity, given by ReLU(x) = max(x,0)

The CNN architecture chosen to perform SSR in this study was based on a residual U-Net. This architecture has been previously shown to be robust in many applications, such as deep artefact suppression of real-time cine CMR data [10] and ventricular segmentation [11–13]. A residual U-Net is a multi-scale CNN where images are sequentially down-sampled and then up-sampled with the network learning the difference between the input and desired output (residual) rather than the desired output directly [14]. In a residual U-Net, the learnt residual is added to the input data to produce the final output data [15]. In this study, a 3D residual U-Net was trained with paired high-resolution ‘ground truth’ data and corresponding synthetic low-resolution images (Fig. 1). This network structure was chosen for the final implementation as it was found to be more accurate than a conventional U-Net for this application (see Additional file 3). Each convolutional layer had a filter size of 3x3x3 and was equipped with a rectified linear unit as nonlinearity, except the last layer that produced the residual update. We used a smaller network size than the classical U-Net architecture to avoid overfitting and loss of generalizability. The filters were equally weighted in all domains and hence no directions were favoured in the training process. The output of the network was projected to positive numbers by a rectified linear unit to enforce non-negativity.

### Preparation of synthetic training data

The synthetic training data was created from conventional high-resolution WH-bSSFP data (without any obvious artefacts due to breathing or arrhythmia) collected from previously scanned children and adults with paediatric heart disease or congenital heart disease. The training data set contained 500 3D WH-bSSFP images (26 ± 13 years, range: 5–80 years. Male: n= 299. Heart rate: 67 ± 9 bpm, range: 41–86). A full list of diagnoses can be found in Additional file 1. Sequence parameters for the cardiac triggered, respiratory navigated high-resolution WH-bSSFP sequence are shown in Table 1.

**Table 1:** Imaging parameters. Imaging parameters for the training/testing of the network, as well as prospective data

	High-resolution WH-bSSFP	Low-resolution WH-bSSFP
Orientation	Sagittal	Sagittal
Matrix (*kx-ky*)	~ 256 × 144	~ 256 × 72
Acceleration in *ky*	×2 (GRAPPA)	×2 (GRAPPA)
GRAPPA reference lines	24	24
Partial-Fourier in *ky*	6/8	6/8
FOV *x-y* (mm)	~ 400 × 238	~ 400 × 238
Slices	~ 96	~ 48
Slice thickness (mm)	~ 1.6	~ 3.2
Partial-Fourier in *kz*	6/8	6/8
FOV *z* (mm)	~ 154	~ 154
Flip angle (deg)	90^o^	90^o^
TE/TR (ms)	~ 1.6/~ 3.6	~ 1.6/~ 3.6
Bandwidth (Hz/Pixel)	~ 592	~ 592
Lines per segment	~ 30	~ 30
Cardiac triggering	Yes	Yes
Respiratory navigator	Yes (window 3 mm)	Yes (window 3 mm)
Spatial resolution (mm)	~ 1.6 × 1.6 × 1.6	~ 1.6 × 3.2 × 3.2
Temporal resolution (ms)	~ 108	~ 108
Total acquisition time (mins)	~ 8.1 (range: 3.3–14.8)	~ 2.9 (range: 1.1–5.0)

Using these 500 data sets, low-resolution data was created by simulating 50% slice resolution and 50% phase resolution. The first step was to crop/pad the high-resolution data to a 256 × 256 matrix with 96 slices, to make the data consistent for training. This was followed by Fourier transform to produce a synthetic k-space. The outer 50% of k-space in the slice and phase encode direction were then zeroed, simulating two-fold down-sampling of the data in both directions. In addition, 75% partial Fourier in both the slice and phase encoding directions was simulated by further asymmetric zeroing in k-space. The resultant simulated k-space was then inverse Fourier transformed back to image space, and the absolute value taken. This produced the synthetic low-resolution data whilst maintaining a matrix size of 256x256x96. Both the high- and low-resolution whole heart data were further cropped to a 192 × 192 matrix, in all 96 slices, to constrain the learning problem to the anatomy of interest (heart). Finally, each 3D data set was normalized to have signal intensities in the range [0, 1]. All processing required for creation of the synthetic training data was performed in MATLAB (2016b, The MathWorks, Inc., Natick, Massachusetts, USA). A flow diagram of the steps necessary to create the synthetic data is included in Additional file 2.

### Network training and validation

Implementation and training of the U-Net was done in Python with TensorFlow [16]. We minimised the **ℓ**^**1**^-loss of the reconstructed volume to the desired ground truth, as this was found to be more accurate than the **ℓ**^**2**^-loss for this application (see Additional file 3). The training was done for 200 epochs with the Adaptive Moment Estimation algorithm (ADAM) [17], with an initial learning rate of 10^− 3^ and batches of two volumes. The total training time for each network took ~ 38 h on a Titan XP GPU (NVIDIA Corporation Santa Clara, California, USA) with 12Gb memory.

The trained network was validated with synthetic test data created in the same way as the training data. The synthetic test data consisted of 25 previously scanned children and adults with paediatric heart disease or congenital heart disease. These patients were not included in the training data set (27 ± 12 years, range: 10–51. Male: n=13. Heart rate: 69 ± 9 bpm, range: 52–85 . A full list of diagnoses can be found in Additional file 1). The resulting super-resolved data were compared to the ground truth, high-resolution data using mean square error (MSE) and Structural Similarity Index (SSIM).

### Generalisability

The SRR network was specifically trained to super-resolve a given low resolution data set. Therefore, we wanted to assess the robustness of the trained network to inputs with different resolutions of the synthetic down-sampled data. To do this, we used the 25 synthetic test data sets, described above. We simulated resolutions from 10% slice and phase resolution to 100% slice and phase resolution, in increments of 10%. The test data was created as described above, but with varying amount of zeros used in the outer portions of k-space in the slice and phase encode direction. The resulting super-resolved data were compared to the ground truth, high-resolution data using MSE and SSIM. The results of these analyses were averaged over the entire volume for each patient.

### Prospective clinical study

Forty children and adults with paediatric or congenital heart disease referred to our centre for clinical CMR were included in the prospective part of the study during September and October 2019 (27 ± 14 years, range: 11–64. Male: n=20. Heart rate: 68 ± 11 bpm, range: 45–95 . A full list of diagnoses can be found in Additional file 1). Exclusion criteria were: i) Significant metal artefact due to implanted medical devices, and ii) Arrhythmia. All patients were imaged on a 1.5 T CMR scanner (Avanto, Siemens Healthineers, Erlangen, Germany) with vector electrocardiographic (VCG) gating. Low-resolution WH-bSSFP data (spatial resolution; 1.6 × 3.2 × 3.2 mm) and high-resolution WH-bSSFP data (spatial resolution; 1.6 × 1.6 × 1.6 mm) were both acquired with cardiac triggering and respiratory navigation, in all subjects (see Table 1 for acquisition parameters). The trained network was then used to perform super-resolution reconstruction on the low-resolution data to produce data with a spatial resolution of 1.6 × 1.6 × 1.6 mm.

The use of retrospectively collected training and test data, as well as collection of prospective whole heart data was approved by the local research ethics committee, and written consent was obtained from all subjects/guardians (Ref: 06/Q0508/124).

### Analysis of prospective data

Vessel diameters, as well as quantitative and qualitative image quality, were measured on both the low-resolution and super-resolutionWH-bSSFP data and compared to reference standard high-resolution WH-bSSFP data. All measurements were made using in-house plugins for the OsiriX open source DICOM viewing platform (Osirix v.9.0, OsiriX Foundation, Geneva, Switzerland) [18]. For all analysis, the observers were presented with each anonymized data set (including repeated volumes for intra-observer variability) in a randomised order, blinded to diagnosis, patient number and type of sequence.

#### Vessel diameter measurements

Diameters were measured manually by two CMR specialists (M.Q. and A.G.) from multi-planar reformats (MPR’s) of the ascending aorta (AAo), descending aorta (DAo), main pulmonary artery (MPA), right pulmonary artery (RPA), left pulmonary artery (LPA) and proximal left coronary artery (LCA). Each clinician was the primary observer for 20 unique patient data sets, of which 10 were re-evaluated to assess intra-observer variability. In addition, each observer assessed 10 patient data sets from the other primary observer, to evaluate inter-observer variability. Thus, each observer scored and processed 40 patient data sets. Overall 20 patient data sets were used to evaluate intra-observer variability and the other 20 patient data sets used to evaluate inter-observer variability. For each vessel, two perpendicular diameter measurements were made, and the average was used for all further analyses.

#### Diagnostic accuracy and confidence

Identification of abnormal anatomy was performed by three independent clinical observers (M.Q., A.G. and K.M.). Patients were selected from the prospective cohort if they had congenital heart disease, resulting in 21 patients being assessed (27 ± 14 years, range: 11–64. Male: n=20. Heart rate: 68 ± 11 bpm, range: 45–95. A full list of diagnoses can be found in Additional file 1).

Each clinician viewed the high-resolution, low-resolution and super-resolution 3D WH-bSSFP data in a completely randomised order to identify the presence of the following abnormalities: 1) MPA stenosis, 2) RPA stenosis, 3) LPA stenosis, 4) Right coronary artery (RCA) abnormality (course or stenosis), 5) Left coronary artery abnormality (course or stenosis), 6) Coarctation of the Aorta, 7) Abnormal Aortic Arch anatomy (including presence of large aorta-pulmonary collaterals) and 8) Ventricular septal defect. Each abnormality was scored on a 5-point Likert scale (1 = Definitely not present, 2 = Probably not present, 3 = Unclear, 4 = Probably present, 5 = Definitely present), allowing evaluation of both diagnostic accuracy and confidence. For diagnostic accuracy (sensitivity and specificity), scores of 1 and 2 were coded as absent, and 4 and 5 were coded as present. A score of 3 was coded as a misdiagnosis. For diagnostic confidence, scores of 1 and 5 were coded as 2 (high confidence), score 2 and 4 were coded 1 (intermediate confidence) and a score of 3 was coded as 0 (low confidence).

#### Qualitative and quantitative image quality

The MPR data for the great vessels (AAo, DAo, MPA, RPA and LPA) was graded on a 5-point Likert scale in three categories: sharpness of vessel borders (1 = non-diagnostic, 2 = poor, 3 = adequate, 4 = good, 5 = excellent), image distortion (1 = non-diagnostic, 2 = severe, 3 = moderate, 4 = mild, 5 = minimal), and residual artefacts (1 = non-diagnostic, 2 = severe, 3 = moderate, 4 = mild, 5 = minimal).

Vessel edge sharpness (ES) was also calculated from the great vessel MPR’s by measuring the maximum gradient of the normalized pixel intensities across the border of the vessel of interest as previously described [19]. Edge sharpness was calculated in 60 positions around the vessel, and the average value was used for comparison.

Estimated signal-to-noise ratio (eSNR) and estimated contrast-to-noise ratio (eCNR) were assessed in a mid-thoracic slice that included blood pool, ventricular myocardium and lung. eSNR was calculated as the ratio of average blood signal intensity to the average noise signal intensity, taken in the lungs [20]. eCNR was calculated as the ratio of blood signal intensity to average myocardial signal intensity [20].

### Statistics

Statistical analyses were performed by using the R software (Rstudio, v.3.5). Comparisons of continuous variables (vessel diameters, edge sharpness, eSNR and eCNR) across of all three groups was performed using one-way repeated measures analysis of variance (ANOVA) with post hoc testing using Holm correction for significant results. Comparison of Likert data was performed using the Friedman’s test with post-hoc testing using the Nemenyi test for significant results. The Friedman’s test with post-hoc Nemenyi comparisons was also used to compare diagnostic confidence scores. Inter and intra-observer variability was assessed using one-way intraclass correlations (ICC), displayed with their 95% confidence intervals. Comparison of acquisition time between the high-resolution and low-resolution WH-bSSFP sequences was performed using a paired t-test. For assessment of agreement of diameter measurements, the high-resolution WH-bSSFP data was used as the reference standard for Bland-Altman analysis. Sensitivity and specificity were calcaulted and displayed with their 95% confidence intervals. Inter-observer agreement for identification of lesions was assessed using Fleiss’s Kappa. A *p*-value of less than 0.05 indicated a significant difference.

## Results

### Network validation

**Fig. 2:**
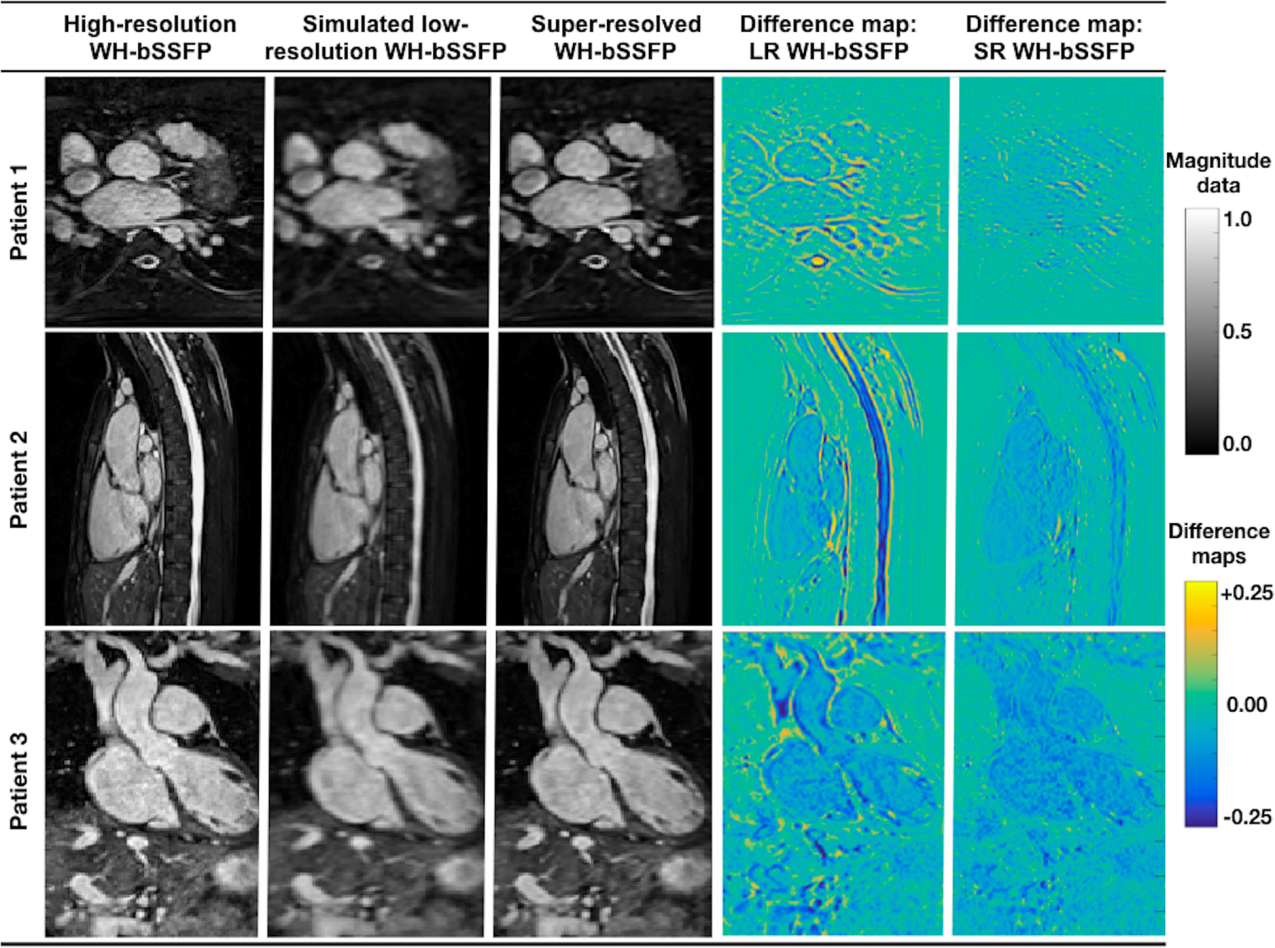
Synthetic test data. Example image quality from the synthetic test data in three patients. Left: Original high-resolution WH-bSSFP data, Middle: Simulated low-resolution WH-bSSFP data, Right: Resulting super-resolved data

Figure 2 shows examples of original high-resolution data, simulated low-resolution data and accompanying super-resolved data. Due to the simulated down-sampling, the low-resolution data had a SSIM of 0.87 ± 0.02, and a MSE of 1.28 ± 0.57 × 10^− 3^, compared to the high-resolution data. After SSR, the SSIM significantly increased (*p* < 0.05) to 0.96 ± 0.01 and the MSE significantly decreased (*p* < 0.05) to 0.68 ± 0.45 × 10^− 3^. This demonstrates that SSR enables recovery of features lost in the low-resolution simulation. Additional file 3 shows the same synthetic tests, as trained with alternate network structures, demonstrating the residual U-net, with an **ℓ**^**1**^-loss function gave the best results.

### Generalisability

**Fig. 3:**
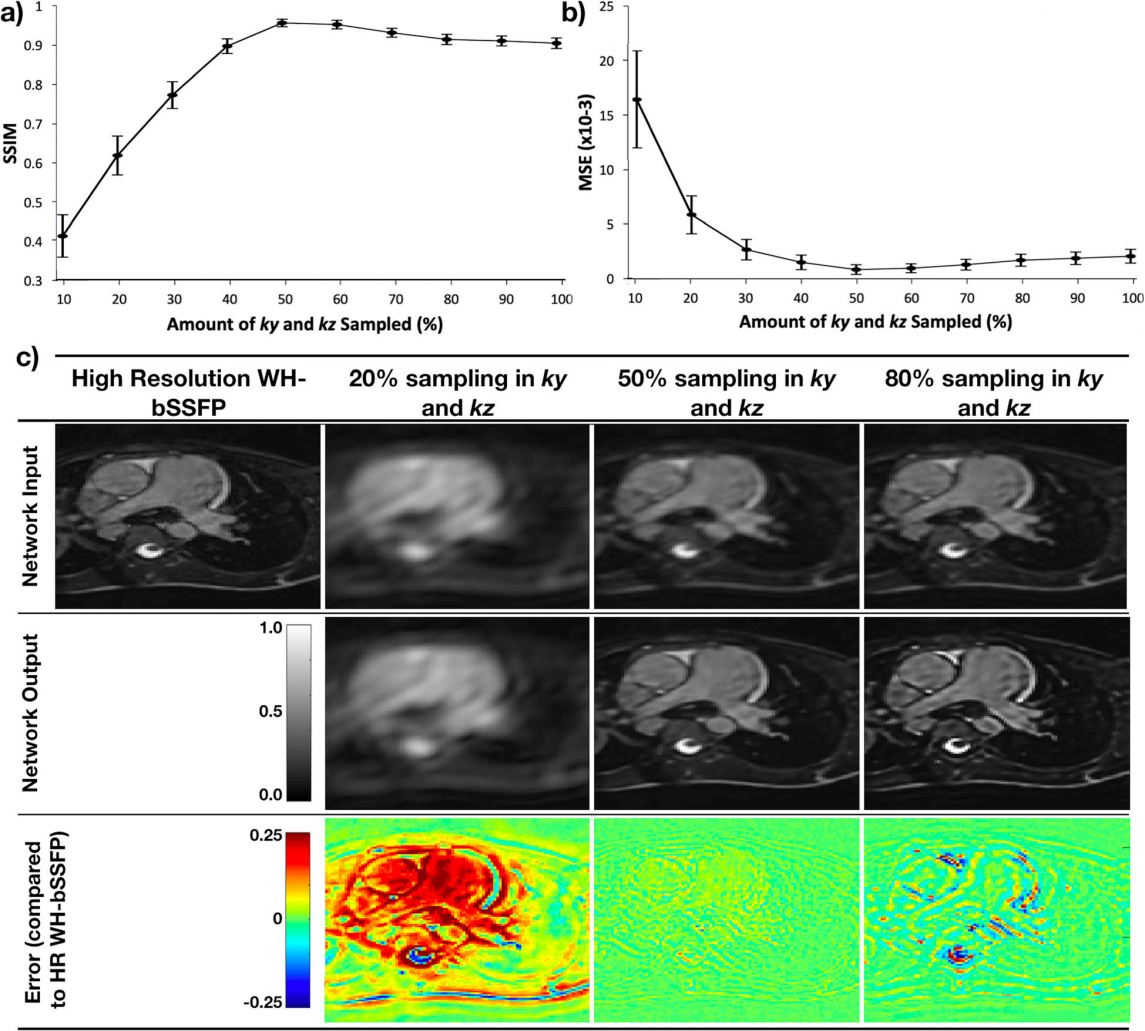
Generalisability tests. Results from the generalisability tests performed on 25 synthetic test data sets. Agreement of super-resolved images with the reference high-resolution WH-bSSFP images at different amounts of down-sampling of the input data; **a** SSIM, **b** MSE. **c** Example low-resolution images at different amounts of down-sampling (input to network), the super-resolved results from the network, and the error maps comparing the super-resolved images to the truth images. See Additional file 4 for full results

Figure 3a and b show that SSIM is highest and MSE is lowest when the input data has the same resolution as the data used for training (50% phase and slice resolution. This can be seen visually in Fig. 3c – at lower resolutions, the network is unable to recover high resolution features resulting in significantly blurred images. At higher resolutions, the network created artificially sharp edges in the resultant images (Additional file 4 shows a table of the results).

### In-vivo study

High-resolution and low-resolution WH-bSSFP data were successfully acquired in all 40 patients. Total acquisition time for high resolution WH data (488 ± 138 s, range: 200 to 889 s) was significantly (*p* < 0.05) higher than the low resolution-WH data (173 ± 54 s, range: 66 to 302 s). The average speed-up in acquisition time was × 2.9 ± 0.8 (range: 1.5 to 5.4).

**Fig. 4:**
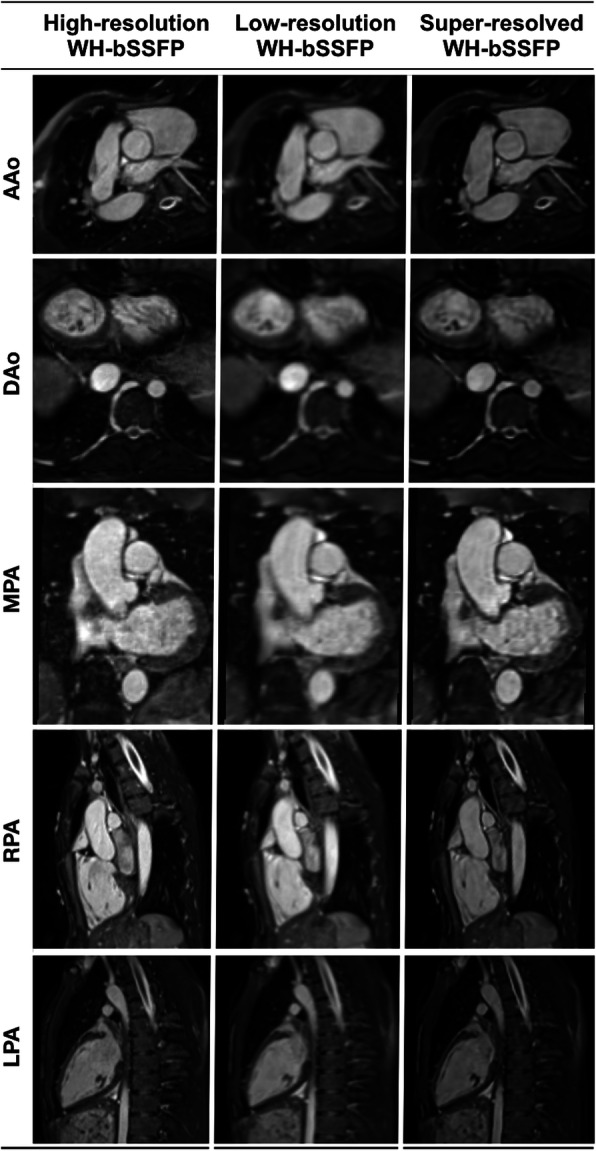
Example images of vessels from the prospective study. Representative image quality from the prospective study. Multi-planar reformats of the ascending aorta (AAo), descending aorta (DAo), main pulmonary artery (MPA), right pulmonary artery (RPA), and left pulmonary artery (LPA), from the high-resolution and low-resolution acquisitions, as well as the super-resolved result

**Fig. 5:**
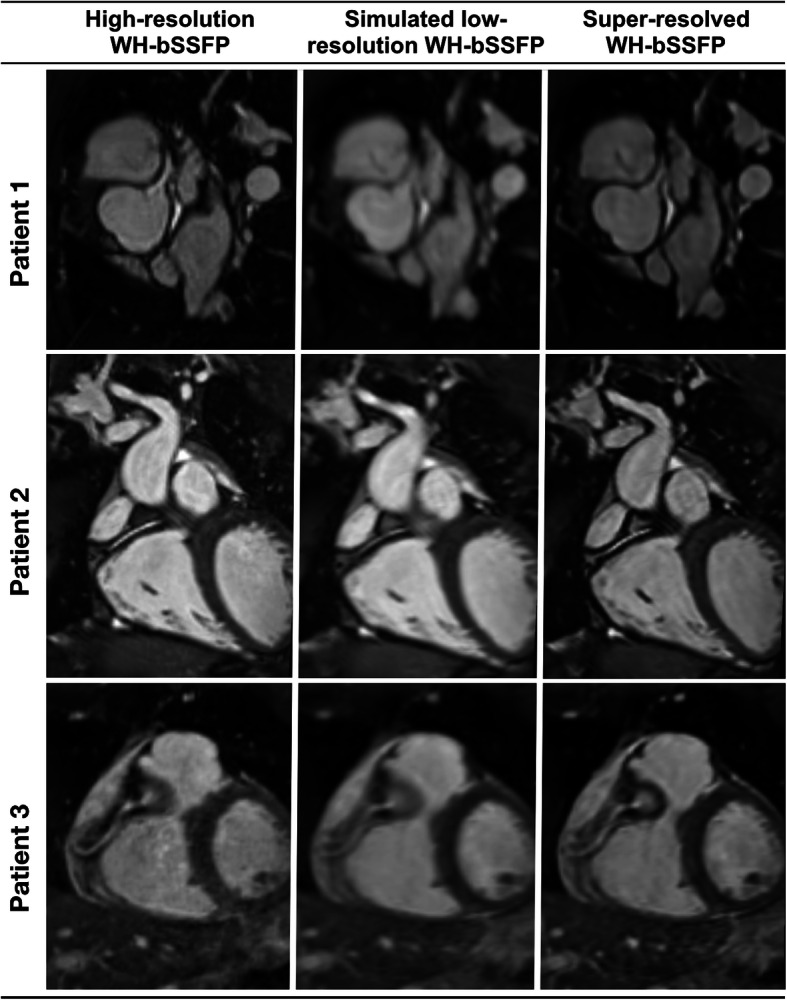
Example images of the coronaries from prospective study. Representative image quality from the prospective study. Multi-planar reformats of the coronary artery from the high-resolution and low-resolution acquisitions, as well as the super-resolved result

SSR was successfully applied to all low-resolution WH-bSSFP data sets. The network took ~ 0.7 s to perform super-resolution per volume (on a Titan XP GPU with 12Gb memory). Representative images are shown in Figs. 4 and 5. It can be seen that image sharpness is improved between the low-resolution data and the super-resolution reconstruction. This is particularly evident in small vessels, such as the coronary arteries (Fig. 5).

#### Quantitative vessel diameter measurements

**Fig. 6:**
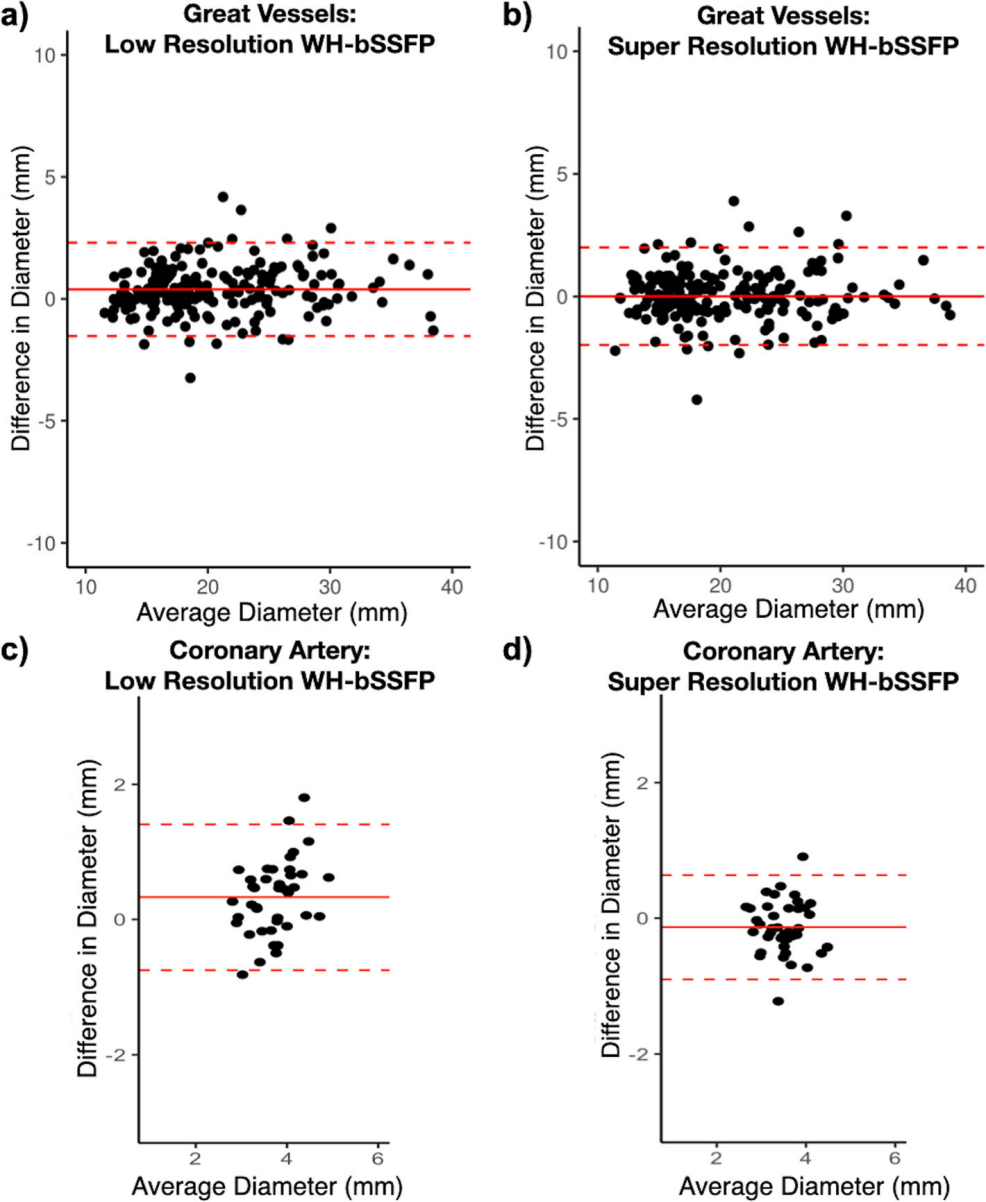
Bland-Altman agreement of vessel diameters. Primary observer; Bland-Altman plots of agreement with high-resolution WH-bSSFP for all vessels; **a** low-resolution WH-bSSFP, **b** super-resolution WH-bSSFP (see Additional file 5 for the Bland-Altman plots of the individual vessels, and for proximal left coronary artery (LCA); **c** low-resolution WH-bSSFP, **d** super-resolution WH-bSSFP.). The solid red line indicates the bias, with the dashed red lines showing the upper and lower limits of agreement (bias±1.96xStandard Deviation) between the techniques

Vessel diameters measured from high-, low- and super-resolution data are shown in Table 2. Figure 6 shows the Bland-Altman plots for all great vessels combined, as well as the Bland-Altman plot for the LCA. The Bland-Altman plots for the individual great vessels shown in Additional file 5. A small but significant overestimation was found in the AAo, DAo, RPA diameters using the low-resolution data compared to the high-resolution data, and a trend for overestimation in the MPA diameter. The proximal left coronary artery diameter measurements also showed a significant overestimation using the low-resolution data compared to the high-resolution data, of 0.3 mm representing ~ 8% overestimation. There were no significant differences between the high-resolution and super-resolution data in the great vessels. However, in the proximal LCA a small but significant underestimation of vessel diameter was seen in the super-resolved data compared to the high-resolution data (of − 0.1 mm, ~ 3%).

**Table 2:** Vessel diameter measurements. Vessel diameter measurements from the prospective patient study (primary observer)

Vessel	n	Mean Diameter ± Standard deviation (mm)			Bland-Altman Analysis^**a**^ Bias (Limits of Agreement)	
		*High-resolution*	*Low-resolution*	*Super-resolution*	*Low-resolution*	*Super-resolution*
AAo	40	28.0 ± 5.6	28.5 ± 5.6*	28.0 ± 5.7^*†*^	0.5 (− 1.7 to 2.6)	−0.1 (−2.2 to 2.1)
DAo	40	17.2 ± 2.7	17.7 ± 3.0*	17.2 ± 2.7^*†*^	0.6 (− 0.8 to 1.9)	0.0 (− 1.1 to 1.2)
MPA	40	24.1 ± 3.7	24.6 ± 3.7	24.4 ± 3.7	0.4 (− 1.8 to 2.7)	0.2 (− 2.1 to 2.6)
RPA	40	16.2 ± 3.3	16.6 ± 3.4*	16.3 ± 3.4^*†*^	0.4 (− 1.2 to 2.0)	0.1 (− 1.9 to 2.1)
LPA	40	17.3 ± 3.2	17.3 ± 3.2	17.0 ± 2.9	0.1 (− 2.0 to 2.1)	−0.2 (− 2.3 to 1.8)
All great vessels	200	20.6 ± 6.0	20.9 ± 6.1	20.6 ± 6.1	0.4 (− 1.5 to 2.3)	0.0 (−2.0 to 2.0)
LCA	40	3.6 ± 0.5	3.9 ± 0.7*	3.4 ± 0.5*	0.3 (− 0.7 to 1.4)	−0.1 (− 0.9 to 0.6)

*AAo* Ascending aorta, *DAo* Descending aorta, *LCA* Left coronary artery, *LPA* Left pulmonary artery, *MPA* Main pulmonary artery, *RPA* Right pulmonar*y* artery

^a^ Bland Altman analysis against the high-resolution WH-bSSFP data. Bias is the mean of the paired difference with the high-resolution WH-bSSFP. Limits of agreements are calculated as bias ± 1.96xSD

*Indicates significant differences with standard high-resolution WH-bSSFP technique as assessed by ANOVA with post hoc testing using Holm correction (*p* < 0.05)

^†^Indicates significant differences with low-resolution WH-bSSFP technique as assessed by ANOVA with post hoc testing using Holm correction (*p* < 0.05)

The inter-observer and intra-observer ICC’s are shown in Table 3. The largely overlapping confidence intervals demonstrated that there were no significant differences in inter-observer and intra-observer variability between any of the techniques in any of the vessels.

**Table 3:** Intra-observer and inter-observer variability. Intra-observer and inter-observer variability; Intra-class correlations for vessel diameters measured from high-resolution, low-resolution and super-resolution WH-bSSFP data. Displayed as ICC (95% confidence intervals)

	High-resolution ICC	Low-resolution ICC	Super-resolution ICC
*Intra-observer variability*			
AAo	0.99 (0.99 to 1.00)	0.99 (0.97 to 0.99)	0.99 (0.98 to 1.00)
DAo	0.99 (0.99 to 1.00)	0.98 (0.95 to 0.99)	0.99 (0.97 to 0.99)
MPA	0.96 (0.92 to 0.98)	0.97 (0.93 to 0.99)	0.97 (0.93 to 0.98)
RPA	0.99 (0.97 to 0.99)	0.99 (0.98 to 1.00)	0.99 (0.99 to 1.00)
LPA	0.98 (0.97 to 0.99)	0.98 (0.96 to 0.99)	0.98 (0.93 to 0.99)
LCA	0.89 (0.74 to 0.95)	0.76 (0.49 to 0.90)	0.89 (0.75 to 0.96)
*Inter-observer variability*			
AAo	0.97 (0.93 to 0.99)	0.96 (0.90 to 0.98)	0.97 (0.94 to 0.99)
DAo	0.84 (0.64 to 0.93)	0.71 (0.41 to 0.87)	0.85 (0.66 to 0.94)
MPA	0.93 (0.84 to 0.97)	0.94 (0.86 to 0.98)	0.92 (0.82 to 0.97)
RPA	0.78 (0.53 to 0.91)	0.53 (0.13 to 0.78)	0.75 (0.47 to 0.89)
LPA	0.87 (0.71 to 0.95)	0.78 (0.52 to 0.90)	0.85 (0.66 to 0.94)
LCA	0.81 (0.59 to 0.92)	0.84 (0.64 to 0.93)	0.88 (0.72 to 0.95)

#### Diagnostic accuracy and confidence

The sensitivities and specificities for detection of any lesion were similar in the high resolution -WH (sensitivity: 0.74, CI: 0.63 to 0.83, specificity: 0.94, CI: 0.91 to 0.96), low resolution WH (sensitivity: 0.71, CI: 0.61 to 0.81, specificity: 0.86, CI: 0.83 to 0.90) and super resolution -WH (sensitivity: 0.73, CI: 0.62 to 0.82, specificity: 0.91, CI: 0.88 to 0.94), with largely overlapping confidence intervals. This was also true for each individual lesion (see Additional file 6). In addition, there was no significant difference the detection of lesions between observers (kappa = 0.15 / 0.09 / 0.13 for HR- WH, LR- WH and SR respectively, *p* > 0.05). See Additional file 6 for individual lesions.

The highest confidence was found with high resolution WH (1.84 ± 0.44), followed by super resolution WH (1.74 ± 0.56) andlow resolution WH (1.59 ± 0.66). The difference between high resolution WH and super resolutionWH was not significant (*p* = 0.2), however there was a significant difference between the high resolution WH and low resolution WH data (*p* = 1.1 × 10^− 6^) and between super resolution WH and low resolution WH (*p* = 0.002).

#### Image quality

Quantitative and qualitative image quality results can be seen in Table 4. Qualitatively, the low-resolution data was found to have significantly lower sharpness of vessel boarders and more image distortion than the high-resolution data, with no significant difference in residual artefacts. After super-resolution reconstruction, there were no significant differences in terms of qualitative image quality with the high-resolution data. However, a significant improvement was seen in terms of sharpness of vessel boarders and image distortion compared to the low-resolution data.

**Table 4:** Qualitative image scores and quantitative image quality results, from the prospective patient study. Displayed as mean ± standard deviation

	n	High-resolution	Low-resolution	Super-resolution
*Qualitative Image Quality Scores*				
Sharpness of vessel borders	600	4.1 ± 0.6	3.1 ± 0.7*	4.2 ± 0.6^†^
Image distortion	600	4.1 ± 0.5	3.8 ± 0.5*	4.0 ± 0.5^†^
Residual artifacts	600	3.8 ± 0.6	3.7 ± 0.6	3.8 ± 0.5
*Quantitative Image Quality Scores*				
eSNR	40	17.2 ± 6.5	22.8 ± 7.3**	27.3 ± 11.1**^,††^
eCNR	40	3.0 ± 0.5	3.2 ± 0.4**	3.3 ± 0.4**
Edge sharpness (mm^−1^)	200	0.8 ± 0.4	0.6 ± 0.3**	1.3 ± 0.7**^,††^

*eCNR* Estimated contrast-to-noise ratio, *eSNR* Estimated signal to noise ratio

* Indicates significant differences with high-resolution WH-bSSFP technique (*p* < 0.05) as assessed by Friedman’s test with post-hoc testing using the Nemenyi test (Qualitative scoring)

^†^ Indicates significant differences with low-resolution WH-bSSFP technique (*p* < 0.05) as assessed by Friedman’s test with post-hoc testing using the Nemenyi test (Qualitative scoring)

** Indicates significant differences with high-resolution WH-bSSFP technique (*p* < 0.05) ANOVA with post hoc testing using Holm correction (Quantitative scoring)

^††^ Indicates significant differences with low-resolution WH-bSSFP technique (*p* < 0.05) ANOVA with post hoc testing using Holm correction (Qualitative scoring)

Quantitative analysis showed that the edge sharpness of the low-resolution data was significantly worse than the high-resolution. After super-resolution, the edge sharpness was significantly better than either the low-resolution or high-resolution data. The eSNR of the low-resolution data was significantly higher than the high-resolution data. After super-resolution, the eSNR improved again, to become significantly higher than either the low-resolution or high-resolution data. The eCNR of the three techniques was similar, however the high-resolution technique was found to have be significantly lower than the low-resolution or super-resolution images.

## Discussion

The main findings of this study were: i) It is possible to train a 3D residual U-Net to perform single volume SRR on synthetically down-sampled WH-bSSFP data, ii) The accuracy of the network is dependent on the input resolution matching that of the training data, iii) SRR of clinically acquired actual low-resolution WH-bSSFP data was successful using the residual U-Net trained using synthetic data, iv) Super-resolution data had better image quality than acquired low resolution data and was comparable to reference standard high-resolution data, v) Vessel diameter measurements made using super-resolved data were not significantly different from reference high-resolution data in the great vessels, but a small underestimation was seen in the coronaries.

### Super-resolution reconstruction

The main benefit of SRR is that it can be applied as a post-processing step and therefore, requires no significant sequence modifications. However, conventional SRR are often computationally intensive and fail to properly recover high resolution features [21, 22]. Recently, deep learning has been used to overcome these problems for a range of imaging problems including brain and body MRI [23, 24]. In this study, we have developed a deep learning framework for super-resolution of 3D WH-bSSFP data. This was done to speed up acquisition of this time-consuming element of many congenital heart disease CMR protocols.

The main requirement for deep learning is paired input and output data that can be used to train the network. Often this must be prospectively acquired, restricting the ability to quickly develop deep-learning platforms. However, simulating low-resolution data is relatively trivial. Thus, synthetic training data can be easily created from previously acquired high-resolution data, allowing rapid development of this framework. A further advantage of using synthetic data is that the ground truth is known, which allows quantitative evaluation of reconstruction accuracy through measurement of SSIM and MSE. Using these metrics, we were able to show that our network successfully recovers high resolution features from previously unseen synthetic low-resolution data. We also showed that the accuracy of our SRR was highly dependent on the resolution of the input data.

### In-vivo study

Demonstrating reconstruction accuracy on synthetic low-resolution test data is an important first step in framework development. However, for true translation it is vital to test performance on actual clinically acquired low-resolution data. In this study, we successfully used our trained residual U-Net to super-resolve prospectively acquired actual low-resolution WH-bSSFP images. We were able to show that super-resolution reconstruction improved subjective image quality compared to the original low-resolution data. Furthermore, as one might expect, quantitative measures of edge sharpness were higher after super-resolution reconstruction compared to the original low-resolution data. Interestingly, eSNR also increased after super-resolution reconstruction, suggesting that the network had some additional de-noising effects.

An important aspect of this study was the comparison of vessels measurements made from high-, low- and super-resolution WH-bSSFP data. In this study all diameter measurements were performed manually as this is most representative of real clinical workflow. We found that vessel diameters were overestimated using the low-resolution data, presumably as a result of the blurred vessel borders. However, there was no statistical differences in vessel diameter measurements between the super-resolution and reference high-resolution data, except in the LCA where a small but significant underestimation was seen. This suggests that super-resolution reconstruction enabled more accurate vessel measurements to be made from data acquired at low resolution. Importantly, the inter-observer and intra-observer variability of SRR diameter measurements were similar to high-resolution diameter measurements. This is an important finding as it demonstrates reliability, which is vital for clinical translation.

The final aspect of this study was evaluation of diagnostic accuracy and confidence. Interestingly, the sensitivity and specificity for identification of common lesions were similar for high-, low- and super-resolution WH-bSSFP data. However, the diagnostic confidence for low resolution WH-bSSFP was significantly lower than both high- and super-resolution data (which were not statistically different). This suggests that although SRR doesn’t necessarily improve accuracy, it does improve diagnostic confidence. This is clinically important, as higher confidence diagnoses can be acted on without further imaging, optimizing patient pathways.

### Clinical implications

We have shown that it is possible to use deep learning SRR to recover the high-resolution features from low-resolution data. The benefit of acquiring low -resolution data is reduced scan time. In our study, the speed-up in acquisition time between the high-resolution and low-resolution WH-bSSFP was found to be ~× 3.0. It should be noted that the resolution was lowered by × 2 in both the slice and phase encoding directions, and one might expect a 4x speed up. However, in our implementation the number of GRAPPA reference lines was the same in both the high- and low-resolution acquisitions, slightly limiting the achievable acceleration. Nevertheless, the ability to acquire WH-bSSFP data in less than 3 min is still clinically useful. Importantly, this framework does not require complex sequence modifications, as is necessary for non-Cartesian or compressed sensing optimised acquisitions. This means in theory it is vendor non-specific, as SRR be employed as a simple post-processing step. In addition, processing is extremely fast (less than a second per volume) unlike more computationally intensive acceleration techniques, such as compressed sensing. However, we have shown that it is vital that the low-resolution input data matches the synthetically down-sampled data used for training. This currently limits the framework as the way down-sampling is implemented varies between vendor. One solution would be to simply train different networks for different vendor data. Thus, this technique holds the potential to significantly shorten cardiac MRscan times in children.

Further reductions in scan time may be achievable by removing the need for respiratory navigation, however this would result in blurring and loss of resolution in the acquired images due to breathing motion. Machine learning algorithms have recently shown the potential to recover high resolution images from this motion corrupted data [25–27].

### Study limitations

The main limitation was of this study was possible absence of underrepresentation of rarer congenital heart defects in the training data. This could theoretically lead to inaccuracies when the network if exposed ‘novel’ defects. However, we believe that our network architecture does not learn specific anatomies, but rather general features of WH-bSSFP such as contrast and vessel edges. To demonstrate this, we acquired a high-resolution WH-bSSFP data set in the abdomen of one adult. SRR of synthetic low-resolution data showed excellent image quality and recovery of high-resolution features (see Additional file 7). This is despite that fact that the network was trained on cardiac WH-bSSFP data and strongly suggests that our network can accurately reconstruct anatomy not present in the training data set.

Another limitation of our approach was that the training and actual input data consisted of coil combined magnitude images, rather than raw multi-coil complex data. The main benefit of this approach was that previously acquired data that was easily retrievable from a conventional clinical image archive could be used for training. However, the absence of phase data in our approach may prevent optimum image restoration.

A further issue is related to images being normalized prior to super resolution.. This could theoretically lead to problems in the presence of very high signal (i.e. non-supressed fat signal or fluid) due to reduced dynamic range. This was not seen in our prospective study but should be more fully investigated in future studies.

A final limitation of this study was that hyperparameters were not meticulously optimised. We did test various loss functions and compared the conventional U-Net to the residual U-Net. However, we did not investigate changing filter sizes or U-Net depth, as results the of the network were good. However, further optimization may be warranted if lower resolution data, which would require a greater amount of super-resolution, were used as the input.

## Conclusion

This paper demonstrates the potential of using a residual U-Net for SRR of rapidly acquired low-resolution whole heart bSSFP data within a clinical setting. Once the network has been trained, the reconstruction times are very short, making these techniques particularly appealing within busy clinical workflow. We have shown that vessel diameter measurements from images reconstructed using a residual U-Net are not statistically significantly different from the reference standard, high-resolution WH-bSSFP techniques. Thus, we believe that this technique may help speed up whole heart CMR in clinical practice.

## Supplementary information

**Additional file 1.** Full demographic information and patient diagnoses.

**Additional file 2.** Flow diagram showing the steps taken to convert the high-resolution WH-bSSFP data, to synthetic low-resolution WH-bSSFP data used to train/test the residual U-Net.

**Additional file 3.** Synthetic test results from different network structures.

**Additional file 4.** Results from the generalisability tests.

**Additional file 5.** Bland-Altman plots of agreement with high-resolution WH-bSSFP for the individual vessels.

**Additional file 6.** Diagnostic Accuracy and Confidence scoring for all lesions.

**Additional file 7.** Application of the super-resolution network applied to abdominal WH-bSSFP data.

## Data Availability

The datasets used and analyzed during the current study are available from the corresponding author on reasonable request.

## Abbreviations

3D: Three dimensional
AAo: Ascending aorta
ADAM: Adaptive Moment Estimation algorithm
ANOVA: Analysis of variance
bSSFP: Balanced steady state free precession
CMR: Cardiovascular magnetic resonance
CNN: Convolutional neural network
DAo: Descending aorta
eCNR: Estimated contrast-to-noise ratio
ES: Edge sharpness
eSNR: Estimated signal-to-noise ratio
GPU: Graphics processing unit
GRAPPA: GeneRalized Autocalibrating Partial Parallel Acquisition
HR: High-resolution
ICC: Intraclass correlation
LCA: Proximal left coronary artery
LPA: Left pulmonary artery
LR: Low-resolution
MPA: Main pulmonary artery
MPR: Multi-planar reformats
MSE: Mean square error
RCA: Right coronary artery
RPA: Right pulmonary artery
SRR: Super-resolution reconstruction
SSIM: Structural similarity index
VCG: Vector electrocardiographic gating
WH: Whole heart

## References

[1] Greil G, Tandon A, Silva Vieira M, Hussain T (2017). 3D whole heart imaging for congenital heart disease. Front Pediatr.

[2] Barkauskas KJ, Rajiah P, Ashwath R, Hamilton JI, Chen Y, Ma D (2014). Quantification of left ventricular functional parameter values using 3D spiral bSSFP and through-time Non-Cartesian GRAPPA. J Cardiovasc Magn Reson.

[3] Stehning C, Börnert P, Nehrke K, Eggers H, Stuber M (2005). Free-breathing whole-heart coronary MRA with 3D radial SSFP and self-navigated image reconstruction. MRM.

[4] Akçakaya M, Basha TA, Goddu B, Goepfert LA, Kissinger KV, Tarokh V (2011). Low-dimensional-structure self-learning and thresholding: regularization beyond compressed sensing for MRI reconstruction. MRM.

[5] Nam S, Akçakaya M, Basha T, Stehning C, Manning WJ, Tarokh V (2013). Compressed sensing reconstruction for whole-heart imaging with 3D radial trajectories: a graphics processing unit implementation. Magn Reson Med.

[6] Lu Y, Yang R, Zhang J, Zhang C, editors. Super resolution image reconstruction in parallel magnetic resonance imaging. IEEE ICCA 2010: 9–11 June 2010; 2010.

[7] Tang Y, Yan P, Yuan Y, Li X (2011). Single-image super-resolution via local learning. Int J Mach Learn Cybern.

[8] Shi W, Caballero J, Ledig C, Zhuang X, Bai W, Bhatia K (2013). Cardiac image super-resolution with global correspondence using multi-atlas patchmatch.

[9] Van Reeth E, Tham IWK, Tan CH, Poh CL (2012). Super-resolution in magnetic resonance imaging: a review. Concept Magn Reson A.

[10] Hauptmann A, Arridge S, Lucka F, Muthurangu V, Steeden JA (2019). Real-time cardiovascular MR with spatio-temporal artifact suppression using deep learning–proof of concept in congenital heart disease. Magn Reson Med.

[11] Yang D, Huang Q, Axel L, Metaxas D, editors. Multi-component deformable models coupled with 2D-3D U-Net for automated probabilistic segmentation of cardiac walls and blood. 2018 IEEE 15th International Symposium on Biomedical Imaging (ISBI 2018); 2018.

[12] Cong C, Zhang H (2018). Invert-U-Net DNN segmentation model for MRI cardiac left ventricle segmentation. J Eng.

[13] Zheng Q, Delingette H, Duchateau N, Ayache N (2018). 3-D consistent and robust segmentation of cardiac images by deep learning with spatial propagation. IEEE Trans Med Imaging.

[14] Ronneberger O, Fischer P, Brox T, editors. U-Net: convolutional networks for biomedical image segmentation. Medical image computing and computer-assisted intervention; Munich: Springer International Publishing; 2015.

[15] Jin KH, McCann MT, Froustey E, Unser M (2017). Deep convolutional neural network for inverse problems in imaging. IEEE Trans Image Process.

[16] Abadi M, Barham P, Chen J, Chen Z, Davis A, Dean J, Devin M, Ghemawat S, Irving G, Isard M. Tensorflow: A system for large-scale machine learning 12th USENIX Symposium on Operating Systems Design and Implementation (OSDI). Savannah; 2016:265-83.

[17] Kingma DP, Ba J (2014). Adam: a method for stochastic optimization. International conference on learning representations (ICLR).

[18] Rosset A, Spadola L, Ratib O (2004). OsiriX: an open-source software for navigating in multidimensional DICOM images. J Digit Imaging.

[19] Steeden JA, Atkinson D, Hansen MS, Taylor AM, Muthurangu V (2011). Rapid flow assessment of congenital heart disease with high-spatiotemporal-resolution gated spiral phase-contrast MR imaging. Radiology.

[20] Kourtidou S, Jones MR, Moore RA, Tretter JT, Ollberding NJ, Crotty EJ (2019). mDixon ECG-gated 3-dimensional cardiovascular magnetic resonance angiography in patients with congenital cardiovascular disease. J Cardiovasc Magn Reson.

[21] Peled S, Yeshurun Y (2002). Superresolution in MRI—perhaps sometimes. Magn Reson Med.

[22] Scheffler K (2002). Superresolution in MRI?. Magn Reson Med.

[23] Pham C-H, Tor-Díez C, Meunier H, Bednarek N, Fablet R, Passat N (2019). Multiscale brain MRI super-resolution using deep 3D convolutional networks. Comput Med Imaging Graph.

[24] Qiu D, Zhang S, Liu Y, Zhu J, Zheng L. Super-resolution reconstruction of knee magnetic resonance imaging based on deep learning. Comput Methods Prog Biomed. 2019;187:105059. 10.1016/j.cmpb.2019.105059 31582263

[25] Jun L, Ming Y, Jue Z, Xiaoying W (2018). Respiratory motion correction for free-breathing 3D abdominal MRI using CNN-based image registration: a feasibility study. Br J Radiol.

[26] Tamada D, Kromrey M-L, Ichikawa S, Onishi H, Motosugi U (2020). Motion artifact reduction using a convolutional neural network for dynamic contrast enhanced MR imaging of the liver. Magn Reson Med Sci.

[27] Küstner T, Armanious K, Yang J, Yang B, Schick F, Gatidis S (2019). Retrospective correction of motion-affected MR images using deep learning frameworks. Magn Reson Med.

