# Supplementary material for: Rapid whole-heart CMR with single volume super-resolution
Source: J Cardiovasc Magn Reson. 2020 Aug 3;22:56. doi: 10.1186/s12968-020-00651-x (PMC7405461; doi:10.1186/s12968-020-00651-x)
Supplement: Supplementary file 1 — Additional file 1. Full demographic information and patient diagnoses. [file 12968_2020_651_MOESM1_ESM.pdf]

Additional File 1

Full demographic information and patient diagnoses, used for training of the network, in the synthetic test data, and for the prospective study.

	Training Data	Synthetic Test Data	Prospective Data	Diagnostic scoring
Male/Female	299/201	13/12	20/20	8/13
Age (years)	26±13 (range: 5-80)	27±12 (range: 10-51)	27±14 (range: 11-64)	33±15 (range: 13-64)
Heart rate (bpm)	67±9 (range: 41-86)	69±9 (range: 52-85)	68±11 (range: 45-97)	68±11 (range: 45-97)
Diagnosis				
Coarctation of the aorta	57	3	4	4
Tetralogy of Fallot / Double outlet right ventricle / Pulmonary atresia with ventricular septal defect	139	3	5	5
Pulmonary valve disease	33	3	0	0
Aortopathy	81	4	8	0
Transposition of the great arteries	59	1	2	2
Aortic valve disease	20	2	5	0
Shunts	27	1	8	8
Complex Congenital Heart Disease	29	1	2	2
Cardiomyopathy	29	5	5	0
Pulmonary Hypertension	11	1	0	0
Tricuspid valve	15	1	1	0
**TOTAL**	**500**	**25**	**40**	**21**

Additional File 2

Flow diagram showing the steps taken to convert the high-resolution WH-bSSFP data, to synthetic low-resolution WH-bSSFP data used to train/test the residual U-Net.

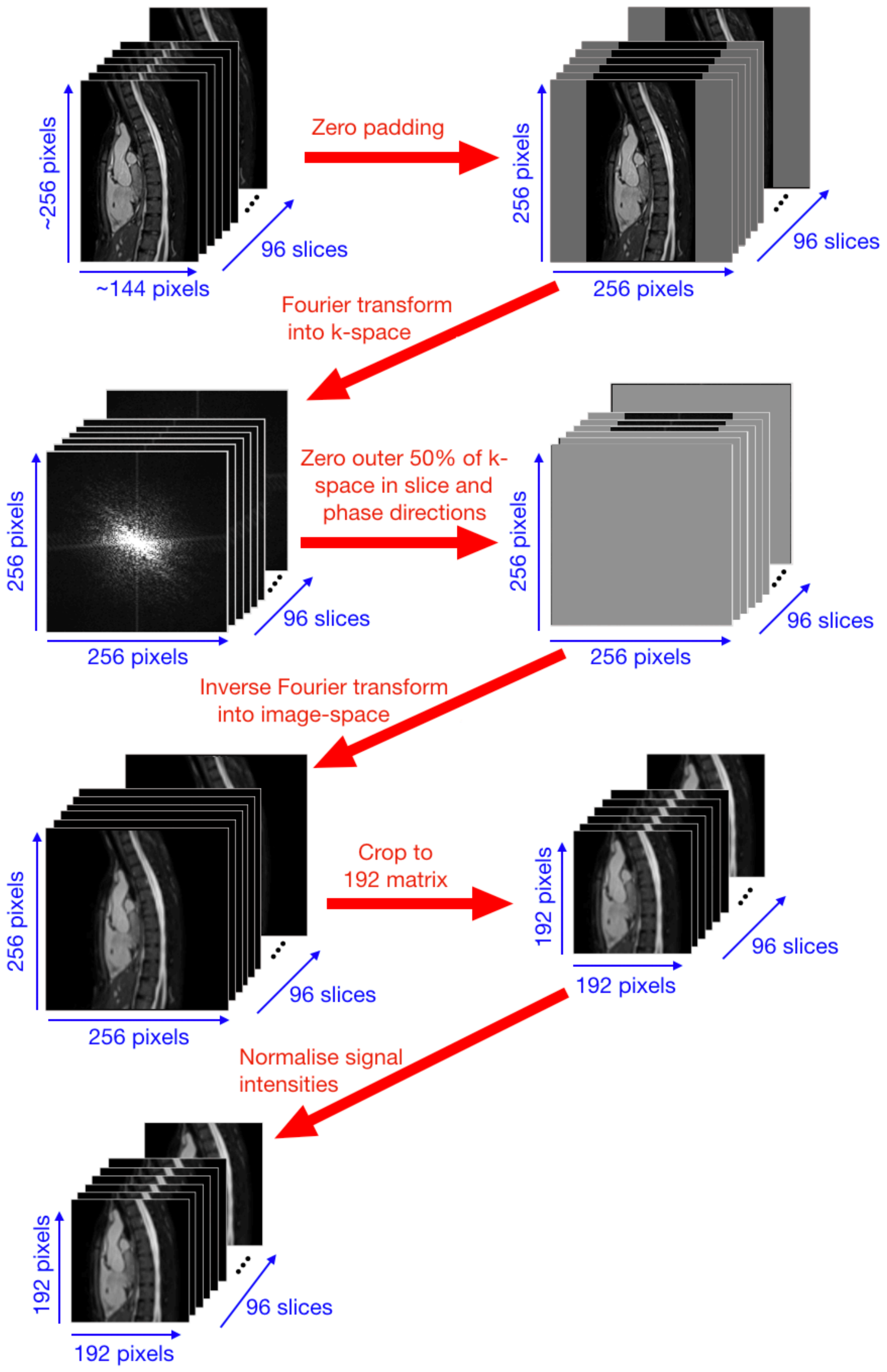

Additional File 3

We investigated two network structures; a U-Net and a residual U-Net, with both a ℓ^1-loss function and an ℓ^2-loss function. For each of the four networks, the

synthetic training data consisted of 500 paired artefact-free 'ground truth' magnitude images and the corresponding low-resolution images, as described in the main paper.

The resulting networks were tested using 25 previously unseen synthetic low-resolution WH-bSSFP data, as described in the main paper. The table below shows the MSE and SSIM results from the different networks, when compared to the reference high-resolution WH-bSSFP data (mean ± standard deviation over the 25 synthetic test data sets).

	**SSIM**	**MSE (x10^{-3})**
Low-resolution data	0.87±0.02	1.28±0.57
Super-resolution data		
• U-Net, ℓ^1-loss	0.94±0.01*	0.71±0.45*
• U-Net, ℓ^2-loss	0.93±0.01*	0.76±0.46*
• Residual U-Net, ℓ^1-loss	0.96±0.01*	0.68±0.45*
• Residual U-Net, ℓ^2-loss	0.94±0.01*	0.68±0.44*

**Indicates statistically significantly poorer result compared to the Residual U-Net, ℓ^1-* loss *($p<0.05$)*

The Residual U-Net with ℓ^1-loss function had significantly higher SSIM (better reconstruction accuracy) than the other networks ($p<0.05$), with significantly lower MSE (better reconstruction accuracy) than the U-Net with ℓ^1-loss or ℓ^2-loss ($p<0.05$). Because of this, the Residual U-Net with an ℓ^1-loss function was chosen in this paper.

Additional File 4

MSE and SSIM results from the generalisability tests, between high-resolution WH-bSSFP and super-resolved data from different levels of down-sampling. Displayed as mean ± standard deviation over the 25 synthetic test data sets.

Percentage of lines Sampled in *ky* and *kz*	SSIM	MSE (x10^{-3})
10%	0.41±0.05*	16.28±4.44*
20%	0.62±0.05*	5.70±1.73*
30%	0.77±0.04*	2.52±0.94*
40%	0.89±0.02*	1.35±0.67*
**50%**	**0.96±0.01**	**0.68±0.45**
60%	0.95±0.01*	0.80±0.41
70%	0.93±0.01*	1.14±0.50*
80%	0.91±0.01*	1.54±0.55*
90%	0.91±0.01*	1.72±0.58*
100%	0.90±0.01*	1.91±0.64*

**Indicates statistically significantly poorer result compared to data sampled with 50% of lines in ky and kz ($p<0.05$)*

Additional File 5

Primary observer; Bland-Altman plots of agreement with high-resolution WH-bSSFP for the individual great vessels; ascending aorta (AAo), descending aorta (DAo), main pulmonary artery (MPA), right pulmonary artery (RPA), and left pulmonary artery (LPA). The solid red line indicates the bias, with the dashed red lines showing the

upper and lower limits of agreement (bias±1.96xStandardDeviation*) between the techniques.*

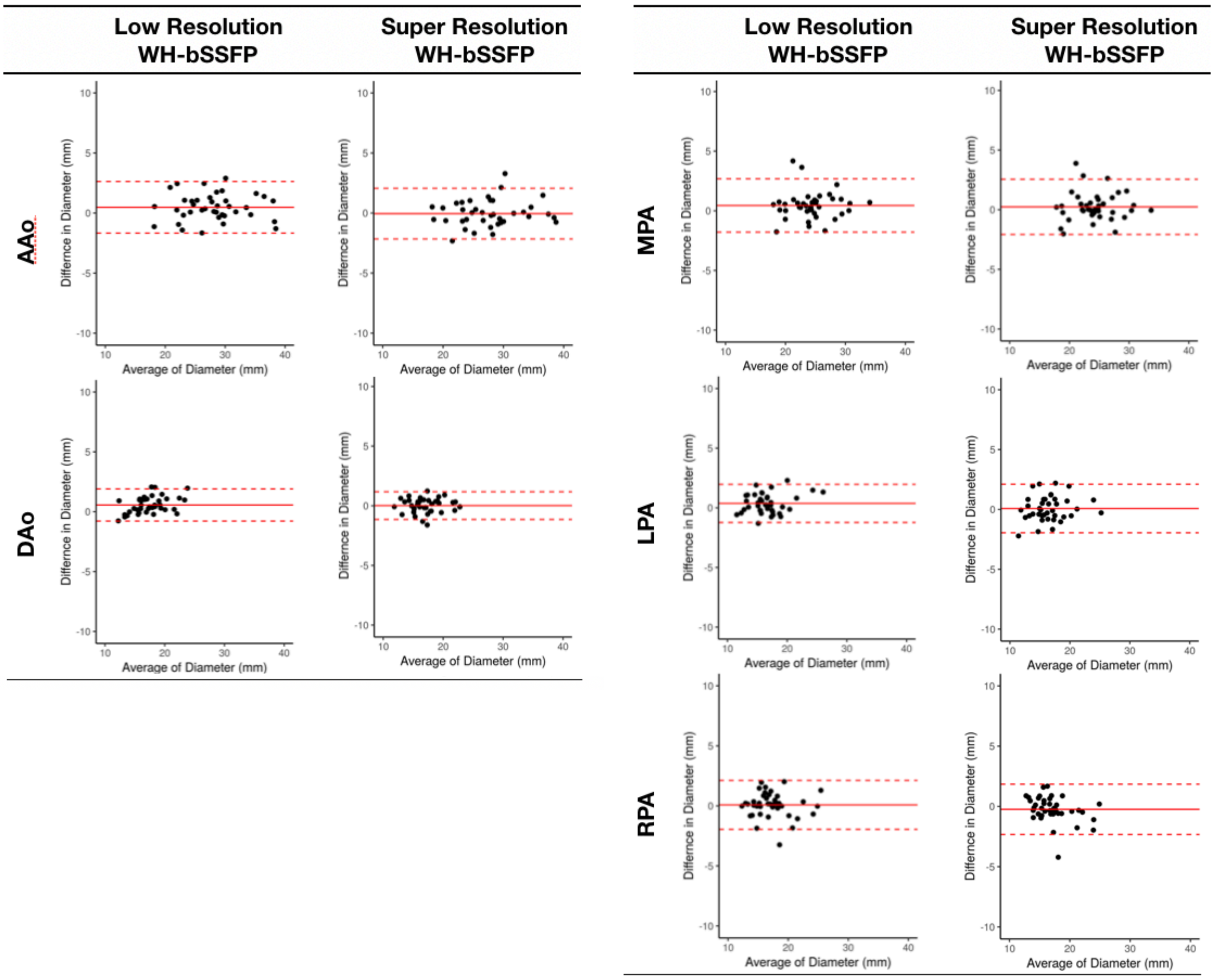

Additional File 6

Diagnostic Accuracy and Confidence from scoring

The table below shows the sensitivities and specificities for detection of the individual lesions in the high-resolution, low-resolution and super-resolution data. Values are displayed as Sensitivity/Specificity (95% confidence intervals).:

MPA; Main Pulmonary Artery

RPA; Right Pulmonary Artery

LPA; Left Pulmonary Artery

RCA; Right Coronary Artery

LCA; Left Coronary Artery

CoA; Coarctation of the Aorta

VSD; Ventricular Septal Defect

	High-resolution	Low-resolution	Super-resolution
**Sensitivity**			
MPA Stenosis	1.00<br>(0.66 to 1.00)	1.00<br>(0.66 to 1.00)	1.00<br>(0.66 to 1.00)
RPA Stenosis	1.00<br>(0.66 to 1.00)	1.00<br>(0.66 to 1.00)	0.89<br>(0.52 to 1.00)
LPA Stenosis	1.00<br>(0.54 to 1.00)	0.83<br>(0.36 to 1.00)	1.00<br>(0.54 to 1.00)
RCA abnormality	0.67<br>(0.22 to 0.96)	0.50<br>(0.12 to 0.88)	0.67<br>(0.22 to 0.96)
LCA abnormality	0.89<br>(0.52 to 1.00)	0.78<br>(0.4 to 0.97)	0.89<br>(0.52 to 1.00)
CoA	0.83<br>(0.36 to 1.00)	0.83<br>(0.36 to 1.00)	0.83<br>(0.36 to 1.00)
Abnormal Aortic Arch	1.00<br>(0.81 to 1.00)	1.00<br>(0.81 to 1.00)	1.00<br>(0.81 to 1.00)
VSD	0.50<br>(0.12 to 0.88)	0.67<br>(0.22 to 0.96)	0.50<br>(0.12 to 0.88)
All abnormalities	0.74<br>(0.63 to 0.83)	0.71<br>(0.61 to 0.81)	0.73<br>(0.62 to 0.82)
**Specificity**			
MPA Stenosis	0.10<br>(0.08 to 0.13)	0.11<br>(0.08 to 1.00)	0.1 0<br>(0.08 to 0.13)
RPA Stenosis	0.11<br>(0.08 to 0.14)	0.11<br>(0.08 to 1.00)	0.11<br>(0.08 to 0.14)
LPA Stenosis	0.10<br>(0.08 to 0.13)	0.10<br>(0.08 to 1.00)	0.10<br>(0.08 to 0.13)
RCA abnormality	0.09<br>(0.07 to 0.12)	0.07<br>(0.05 to 0.88)	0.08<br>(0.06 to 0.11)
LCA abnormality	0.10<br>(0.08 to 0.13)	0.09<br>(0.07 to 0.97)	0.09<br>(0.07 to 0.12)
CoA	0.11<br>(0.09 to 0.15)	0.11<br>(0.08 to 1.00)	0.11<br>(0.09 to 0.15)
Abnormal Aortic Arch	0.08<br>(0.06 to 0.11)	0.08<br>(0.06 to 1.00)	0.08<br>(0.06 to 0.11)

VSD	0.09<br>(0.07 to 0.12)	0.07<br>(0.05 to 0.96)	0.09<br>(0.07 to 0.12)
All abnormalities	0.94<br>(0.91 to 0.96)	0.86<br>(0.83 to 0.90)	0.91<br>(0.88 to 0.94)

The table below shows the inter-rater reliability for the different lesions, as assessed by Friedman's test with post-hoc Nemenyi comparisons. Displayed as ICC (95% confidence intervals).

	**High-resolution**	**Low-resolution**	**Super-resolution**
MPA Stenosis	0.79<br>(0.73 to 0.86)	0.78<br>(0.72 to 0.84)	0.79<br>(0.73 to 0.86)
RPA Stenosis	1.00<br>(0.94 to 1.10)	1.00<br>(0.94 to 1.10)	0.86<br>(0.79 to 0.92)
LPA Stenosis	1.00<br>(0.94 to 1.10)	0.79<br>(0.73 to 0.86)	1.00<br>(0.94 to 1.10)
RCA abnormality	0.56<br>(0.5 to 0.63)	0.29*<br>(0.22 to 0.35)	0.43<br>(0.36 to 0.49)
LCA abnormality	0.59<br>(0.52 to 0.65)	0.44<br>(0.37 to 0.50)	0.53<br>(0.47 to 0.60)
CoA	0.78<br>(0.72 to 0.85)	0.52<br>(0.45 to 0.58)	0.78<br>(0.72 to 0.85)
Abnormal Aortic Arch	0.79<br>(0.73 to 0.85)	0.73<br>(0.67 to 0.80)	0.73<br>(0.66 to 0.79)
VSD	0.21<br>(0.15 to 0.28)	0.39<br>(0.33 to 0.46)	0.22<br>(0.16 to 0.29)
All abnormalities	0.15<br>(0.14 to 0.15)	0.09*<br>(0.08 to 0.10)	0.13<br>(0.12 o 0.14)

** Indicates significant differences between observers (p<0.05) as assessed by Friedman's test with post-hoc testing using the Nemenyi test*

Additionally we assessed diagnostic confidence of the three techniques. High was given by a score of 2, intermediate confidence a score of 1, and low confidence a score of 0. The table below shows mean ± standard deviation of the confidence scores.

	High-resolution	Low-resolution	Super-resolution
MPA Stenosis	2.0 ± 0.2	1.9 ± 0.4	1.9 ± 0.2
RPA Stenosis	2.0 ± 0.1	1.8 ± 0.4*	1.9 ± 0.3†
LPA Stenosis	2.0 ± 0.2	1.8 ± 0.5	1.9 ± 0.3
RCA abnormality	1.6 ± 0.7	1.0 ± 0.9	1.4 ± 0.8
LCA abnormality	1.8 ± 0.5	1.3 ± 0.7	1.5 ± 0.8
CoA	2.0 ± 0.3	1.9 ± 0.4	1.9 ± 0.3
Abnormal Aortic Arch	2.0 ± 0.1	1.9 ± 0.4*	2.0 ± 0.0
VSD	1.5 ± 0.6	1.2 ± 0.7	1.4 ± 0.6
All abnormalities	1.8 ± 0.4	1.6 ± 0.7*	1.7 ± 0.6†

** Indicates significant differences with high-resolution technique (p<0.05) as assessed by Friedman's test with post-hoc testing using the Nemenyi test*

† Indicates significant differences with low-resolution technique (p<0.05) as assessed by Friedman's test with post-hoc testing using the Nemenyi test

Additional File 7

To assess if the large variability and diversity of anatomical variations in congenital heart disease may pose a problem for the networks, we tested the network on a data set which was acquired in the abdomen. This represents anatomy which the network has never seen before.

A Whole-Heart bSSFP acquisition (parameters similar to the high-resolution WH-bSSFP sequence in main text) was acquired in the abdomen, in one adult (Male, 40 years). This data was synthetically down-sampled (as described in the main text), and subsequently super-resolved using the network described in the main text, which had been trained on cardiac data.

For this abdominal data set, the low-resolution images had a MSE of $9.6x10^{-4}$ *compared to the reference high-resolution data, which increased to* $5.0x10^{-4}$ *after super-resolution. Similarly, the SSIM increased from 0.88 to 0.96 after super-resolution reconstruction*.

The figure below shows the image quality of the super-resolution reconstruction in the abdomen.

Although a larger study is needed to validate this properly, this data set suggests that the network does not learn anything about the underlying anatomy, but learns about contrast, edges and vessels. Therefore, the network should be relatively robust to the large range of anatomical variations seen in congenital heart disease.

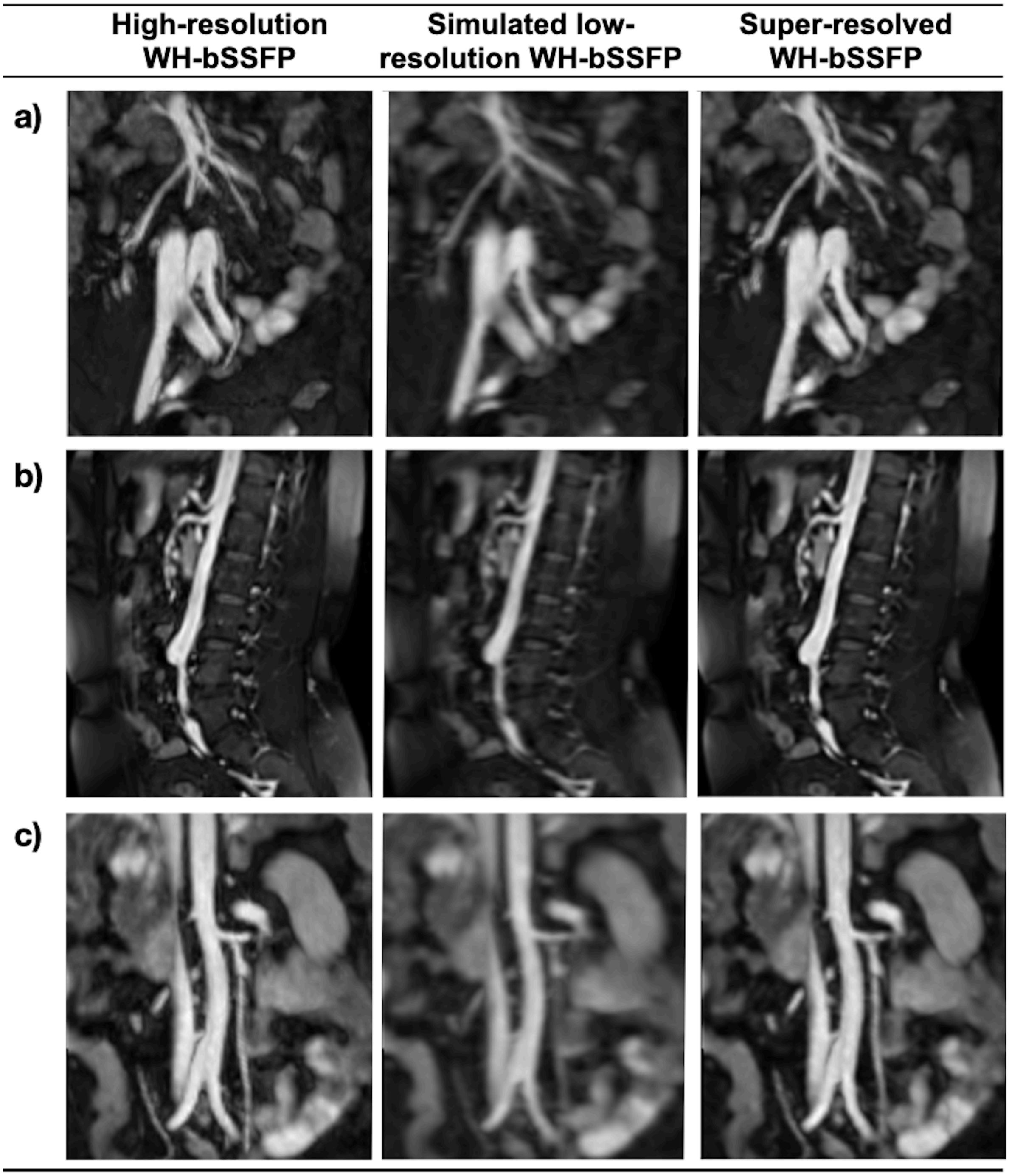

Left: Original high-resolution WH-bSSFP abdominal data, Middle: Simulated low-resolution WH-bSSFP abdominal data, Right: Resulting super-resolved abdominal data. a) anatomy, b) anatomy, c) anatomy.
