# Supplementary material for: Rapid whole-heart CMR with single volume super-resolution
Source: J Cardiovasc Magn Reson. 2020 Aug 3;22:56. doi: 10.1186/s12968-020-00651-x (PMC7405461; doi:10.1186/s12968-020-00651-x)

Additional File 2

Flow diagram showing the steps taken to convert the high-resolution WH-bSSFP data, to synthetic low-resolution WH-bSSFP data used to train/test the residual U-Net.

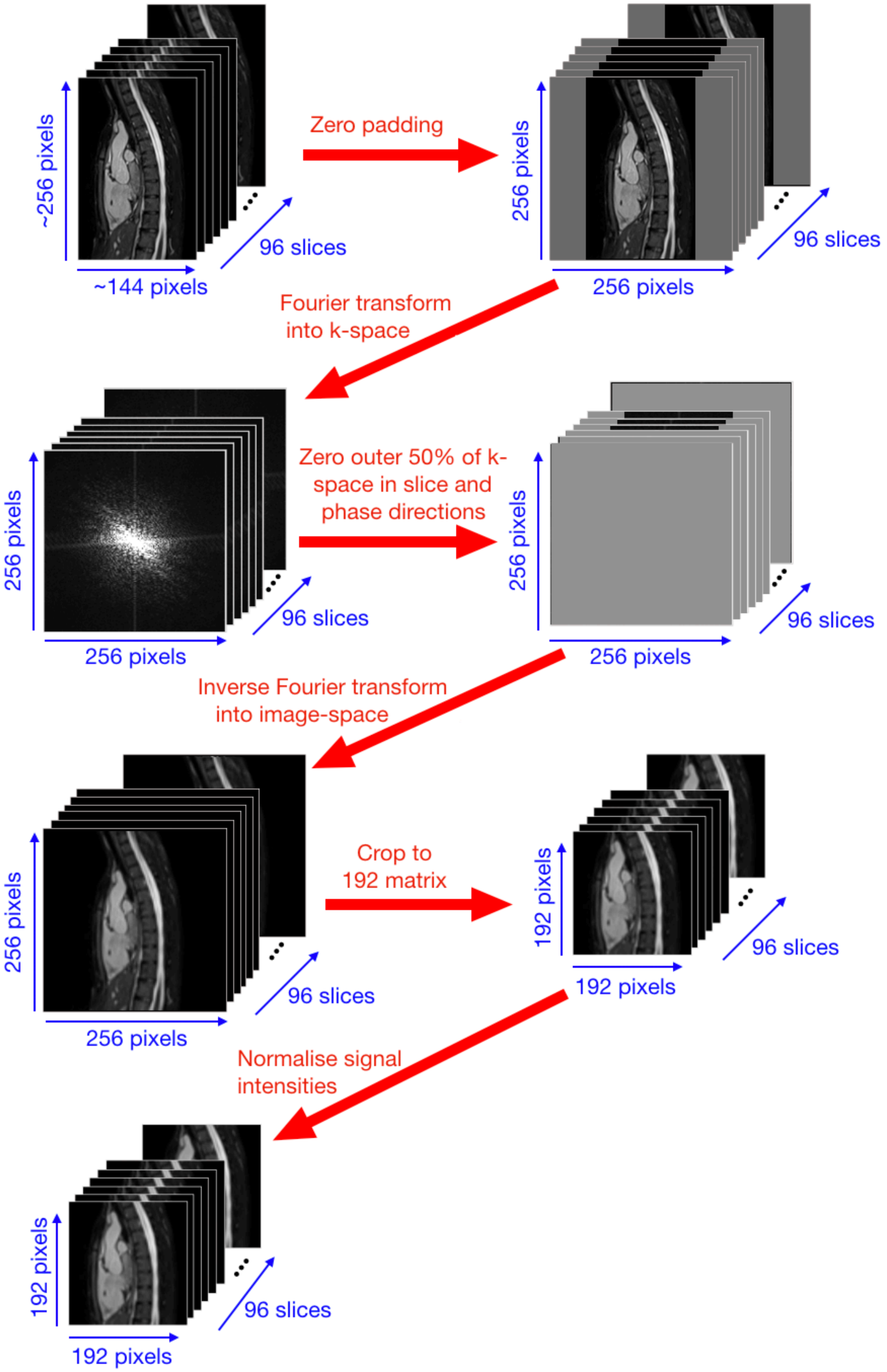

Supplement: Supplementary file 2 — Additional file 2. Flow diagram showing the steps taken to convert the high-resolution WH-bSSFP data, to synthetic low-resolution WH-bSSFP data used to train/test the residual U-Net. [file 12968_2020_651_MOESM2_ESM.pdf]
