# Supplementary material for: Rapid whole-heart CMR with single volume super-resolution
Source: J Cardiovasc Magn Reson. 2020 Aug 3;22:56. doi: 10.1186/s12968-020-00651-x (PMC7405461; doi:10.1186/s12968-020-00651-x)
Supplement: Supplementary file 3 — Additional file 3. Synthetic test results from different network structures. [file 12968_2020_651_MOESM3_ESM.pdf]

Additional File 3

We investigated two network structures; a U-Net and a residual U-Net, with both a ℓ^1-loss function and an ℓ^2-loss function. For each of the four networks, the synthetic training data consisted of 500 paired artefact-free 'ground truth' magnitude images and the corresponding low-resolution images, as described in the main paper.

The resulting networks were tested using 25 previously unseen synthetic low-resolution WH-bSSFP data, as described in the main paper. The table below shows the MSE and SSIM results from the different networks, when compared to the reference high-resolution WH-bSSFP data (mean ± standard deviation over the 25 synthetic test data sets).

	**SSIM**	**MSE ($\times 10^{-3}$)**
Low-resolution data	0.87±0.02	1.28±0.57
Super-resolution data		
• U-Net, ℓ^1-loss	0.94±0.01*	0.71±0.45*
• U-Net, ℓ^2-loss	0.93±0.01*	0.76±0.46*
• Residual U-Net, ℓ^1-loss	0.96±0.01	0.68±0.45
• Residual U-Net, ℓ^2-loss	0.94±0.01*	0.68±0.44

**Indicates statistically significantly poorer result compared to the Residual U-Net with* ℓ^1-loss *($p<0.05$)*

The Residual U-Net with ℓ^1-loss function had significantly higher SSIM (better reconstruction accuracy) than the other networks ($p<0.05$), with significantly lower MSE (better reconstruction accuracy) than the U-Net with ℓ^1-loss or ℓ^2-loss ($p<0.05$). Because of this, the Residual U-Net with an ℓ^1-loss function was chosen in this paper.
