# Supplementary material for: Rapid whole-heart CMR with single volume super-resolution
Source: J Cardiovasc Magn Reson. 2020 Aug 3;22:56. doi: 10.1186/s12968-020-00651-x (PMC7405461; doi:10.1186/s12968-020-00651-x)
Supplement: Supplementary file 4 — Additional file 4. Results from the generalisability tests. [file 12968_2020_651_MOESM4_ESM.pdf]

Additional File 4

MSE and SSIM results from the generalisability tests, between high-resolution WH-bSSFP and super-resolved data from different levels of down-sampling. Displayed as mean ± standard deviation over the 25 synthetic test data sets.

Percentage of lines Sampled in *ky* and *kz*	SSIM	MSE ($x10^{-3}$)
10%	0.41±0.05*	16.28±4.44*
20%	0.62±0.05*	5.70±1.73*
30%	0.77±0.04*	2.52±0.94*
40%	0.89±0.02*	1.35±0.67*
**50%**	**0.96±0.01**	**0.68±0.45**
60%	0.95±0.01*	0.80±0.41
70%	0.93±0.01*	1.14±0.50*
80%	0.91±0.01*	1.54±0.55*
90%	0.91±0.01*	1.72±0.58*
100%	0.90±0.01*	1.91±0.64*

**Indicates statistically significantly poorer result compared to data sampled with 50% of lines in ky and kz ($p<0.05$)*
