# Supplementary material for: Rapid whole-heart CMR with single volume super-resolution
Source: J Cardiovasc Magn Reson. 2020 Aug 3;22:56. doi: 10.1186/s12968-020-00651-x (PMC7405461; doi:10.1186/s12968-020-00651-x)
Supplement: Supplementary file 5 — Additional file 5. Bland-Altman plots of agreement with high-resolution WH-bSSFP for the individual vessels. [file 12968_2020_651_MOESM5_ESM.pdf]

Additional File 5

Primary observer; Bland-Altman plots of agreement with high-resolution WH-bSSFP for the individual vessels; ascending aorta (AAo), descending aorta (DAo), main pulmonary artery (MPA), right pulmonary artery (RPA), and left pulmonary artery (LPA)*. The solid red line indicates the bias, with the dashed red lines showing the upper and lower limits of agreement (bias*±1.96xStandardDeviation*) between the techniques.*

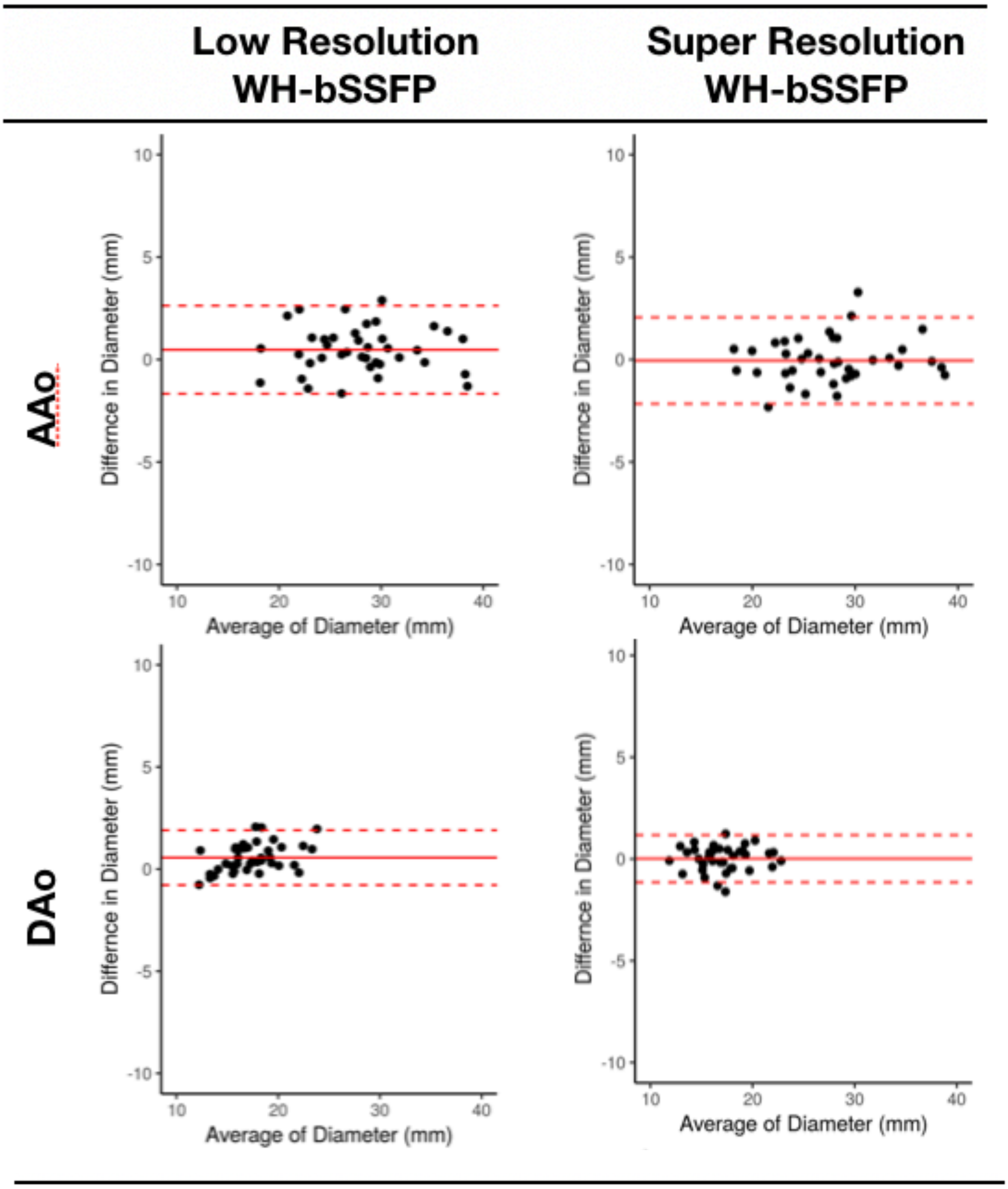

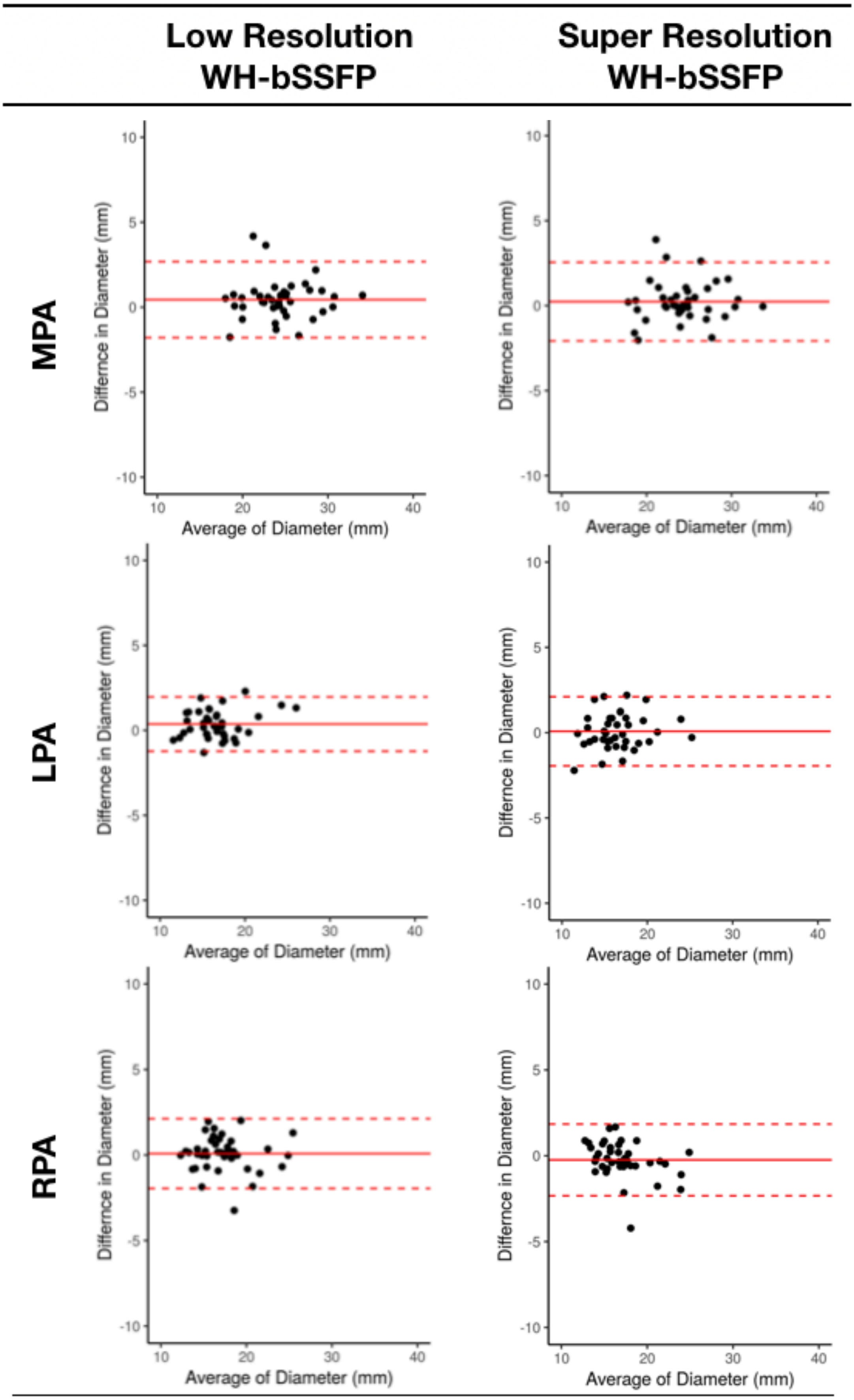
Low Resolution WH-bSSFP
Super Resolution WH-bSSFP
MPA
LPA
RPA
Differnce in Diameter (mm)
Average of Diameter (mm)
10
5
0
-5
-10
10
20
30
40
