# Supplementary material for: Rapid whole-heart CMR with single volume super-resolution
Source: J Cardiovasc Magn Reson. 2020 Aug 3;22:56. doi: 10.1186/s12968-020-00651-x (PMC7405461; doi:10.1186/s12968-020-00651-x)
Supplement: Supplementary file 6 — Additional file 6. Diagnostic Accuracy and Confidence scoring for all lesions. [file 12968_2020_651_MOESM6_ESM.pdf]

Additional File 6

Diagnostic Accuracy and Confidence from scoring

The table below shows the sensitivities and specificities for detection of the individual lesions in the high-resolution, low-resolution and super-resolution data. Values are displayed as Sensitivity/Specificity (95% confidence intervals).:

MPA; Main Pulmonary Artery

RPA; Right Pulmonary Artery

LPA; Left Pulmonary Artery

RCA; Right Coronary Artery

LCA; Left Coronary Artery

CoA; Coarctation of the Aorta

VSD; Ventricular Septal Defect

	High-resolution	Low-resolution	Super-resolution
**Sensitivity**			
MPA Stenosis	1.00<br>(0.66 to 1.00)	1.00<br>(0.66 to 1.00)	1.00<br>(0.66 to 1.00)
RPA Stenosis	1.00<br>(0.66 to 1.00)	1.00<br>(0.66 to 1.00)	0.89<br>(0.52 to 1.00)
LPA Stenosis	1.00<br>(0.54 to 1.00)	0.83<br>(0.36 to 1.00)	1.00<br>(0.54 to 1.00)
RCA abnormality	0.67<br>(0.22 to 0.96)	0.50<br>(0.12 to 0.88)	0.67<br>(0.22 to 0.96)
LCA abnormality	0.89<br>(0.52 to 1.00)	0.78<br>(0.4 to 0.97)	0.89<br>(0.52 to 1.00)
CoA	0.83<br>(0.36 to 1.00)	0.83<br>(0.36 to 1.00)	0.83<br>(0.36 to 1.00)
Abnormal Aortic Arch	1.00<br>(0.81 to 1.00)	1.00<br>(0.81 to 1.00)	1.00<br>(0.81 to 1.00)
VSD	0.50<br>(0.12 to 0.88)	0.67<br>(0.22 to 0.96)	0.50<br>(0.12 to 0.88)
All abnormalities	0.74<br>(0.63 to 0.83)	0.71<br>(0.61 to 0.81)	0.73<br>(0.62 to 0.82)
**Specificity**			
MPA Stenosis	0.10<br>(0.08 to 0.13)	0.11<br>(0.08 to 1.00)	0.1 0<br>(0.08 to 0.13)
RPA Stenosis	0.11<br>(0.08 to 0.14)	0.11<br>(0.08 to 1.00)	0.11<br>(0.08 to 0.14)
LPA Stenosis	0.10<br>(0.08 to 0.13)	0.10<br>(0.08 to 1.00)	0.10<br>(0.08 to 0.13)
RCA abnormality	0.09<br>(0.07 to 0.12)	0.07<br>(0.05 to 0.88)	0.08<br>(0.06 to 0.11)
LCA abnormality	0.10<br>(0.08 to 0.13)	0.09<br>(0.07 to 0.97)	0.09<br>(0.07 to 0.12)
CoA	0.11<br>(0.09 to 0.15)	0.11<br>(0.08 to 1.00)	0.11<br>(0.09 to 0.15)
Abnormal Aortic Arch	0.08<br>(0.06 to 0.11)	0.08<br>(0.06 to 1.00)	0.08<br>(0.06 to 0.11)
VSD	0.09<br>(0.07 to 0.12)	0.07<br>(0.05 to 0.96)	0.09<br>(0.07 to 0.12)
All abnormalities	0.94<br>(0.91 to 0.96)	0.86<br>(0.83 to 0.90)	0.91<br>(0.88 to 0.94)

The table below shows the inter-rater reliability for the different lesions, as assessed by Fleiss's Kappa comparisons. Displayed as Kappa (95% confidence intervals).

	**High-resolution**	**Low-resolution**	**Super-resolution**
MPA Stenosis	0.79 (0.73 to 0.86)	0.78 (0.72 to 0.84)	0.79 (0.73 to 0.86)
RPA Stenosis	1.00 (0.94 to 1.10)	1.00 (0.94 to 1.10)	0.86 (0.79 to 0.92)
LPA Stenosis	1.00 (0.94 to 1.10)	0.79 (0.73 to 0.86)	1.00 (0.94 to 1.10)
RCA abnormality	0.56 (0.5 to 0.63)	0.29* (0.22 to 0.35)	0.43 (0.36 to 0.49)
LCA abnormality	0.59 (0.52 to 0.65)	0.44 (0.37 to 0.50)	0.53 (0.47 to 0.60)
CoA	0.78 (0.72 to 0.85)	0.52 (0.45 to 0.58)	0.78 (0.72 to 0.85)
Abnormal Aortic Arch	0.79 (0.73 to 0.85)	0.73 (0.67 to 0.80)	0.73 (0.66 to 0.79)
VSD	0.21 (0.15 to 0.28)	0.39 (0.33 to 0.46)	0.22 (0.16 to 0.29)
All abnormalities	0.15 (0.14 to 0.15)	0.09* (0.08 to 0.10)	0.13 (0.12 o 0.14)

** Indicates significant differences between observers ($p<0.05$)*

Additionally we assessed diagnostic confidence of the three techniques. High was given by a score of 2, intermediate confidence a score of 1, and low confidence a score of 0. The table below shows mean ± standard deviation of the confidence scores.

	High-resolution	Low-resolution	Super-resolution
MPA Stenosis	2.0 ± 0.2	1.9 ± 0.4	1.9 ± 0.2
RPA Stenosis	2.0 ± 0.1	1.8 ± 0.4*	1.9 ± 0.3†
LPA Stenosis	2.0 ± 0.2	1.8 ± 0.5	1.9 ± 0.3
RCA abnormality	1.6 ± 0.7	1.0 ± 0.9	1.4 ± 0.8
LCA abnormality	1.8 ± 0.5	1.3 ± 0.7	1.5 ± 0.8
CoA	2.0 ± 0.3	1.9 ± 0.4	1.9 ± 0.3
Abnormal Aortic Arch	2.0 ± 0.1	1.9 ± 0.4*	2.0 ± 0.0
VSD	1.5 ± 0.6	1.2 ± 0.7	1.4 ± 0.6
All abnormalities	1.8 ± 0.4	1.6 ± 0.7*	1.7 ± 0.6†

** Indicates significant differences with high-resolution technique (p<0.05) as assessed by Friedman's test with post-hoc testing using the Nemenyi test*

† Indicates significant differences with low-resolution technique (p<0.05) as assessed by Friedman's test with post-hoc testing using the Nemenyi test
