# Supplementary material for: Rapid whole-heart CMR with single volume super-resolution
Source: J Cardiovasc Magn Reson. 2020 Aug 3;22:56. doi: 10.1186/s12968-020-00651-x (PMC7405461; doi:10.1186/s12968-020-00651-x)
Supplement: Supplementary file 7 — Additional file 7. Application of the super-resolution network applied to abdominal WH-bSSFP data. [file 12968_2020_651_MOESM7_ESM.pdf]

Additional File 7

To assess if the large variability and diversity of anatomical variations in congenital heart disease may pose a problem for the networks, we tested the network on a data set which was acquired in the abdomen. This represents anatomy which the network has never seen before.

A Whole-Heart bSSFP acquisition (parameters similar to the high-resolution WH-bSSFP sequence in main text) was acquired in the abdomen, in one adult (Male, 40 years). This data was synthetically down-sampled (as described in the main text), and subsequently super-resolved using the network described in the main text, which had been trained on cardiac data.

For this abdominal data set, the low-resolution images had a MSE of $9.6x10^{-4}$ *compared to the reference high-resolution data, which increased to* $5.0x10^{-4}$ *after super-resolution. Similarly, the SSIM increased from 0.88 to 0.96 after super-resolution reconstruction*.

The figure below shows the image quality of the super-resolution reconstruction in the abdomen.

Although a larger study is needed to validate this properly, this data set suggests that the network does not learn anything about the underlying anatomy, but learns about contrast, edges and vessels. Therefore, the network should be relatively robust to the large range of anatomical variations seen in congenital heart disease.

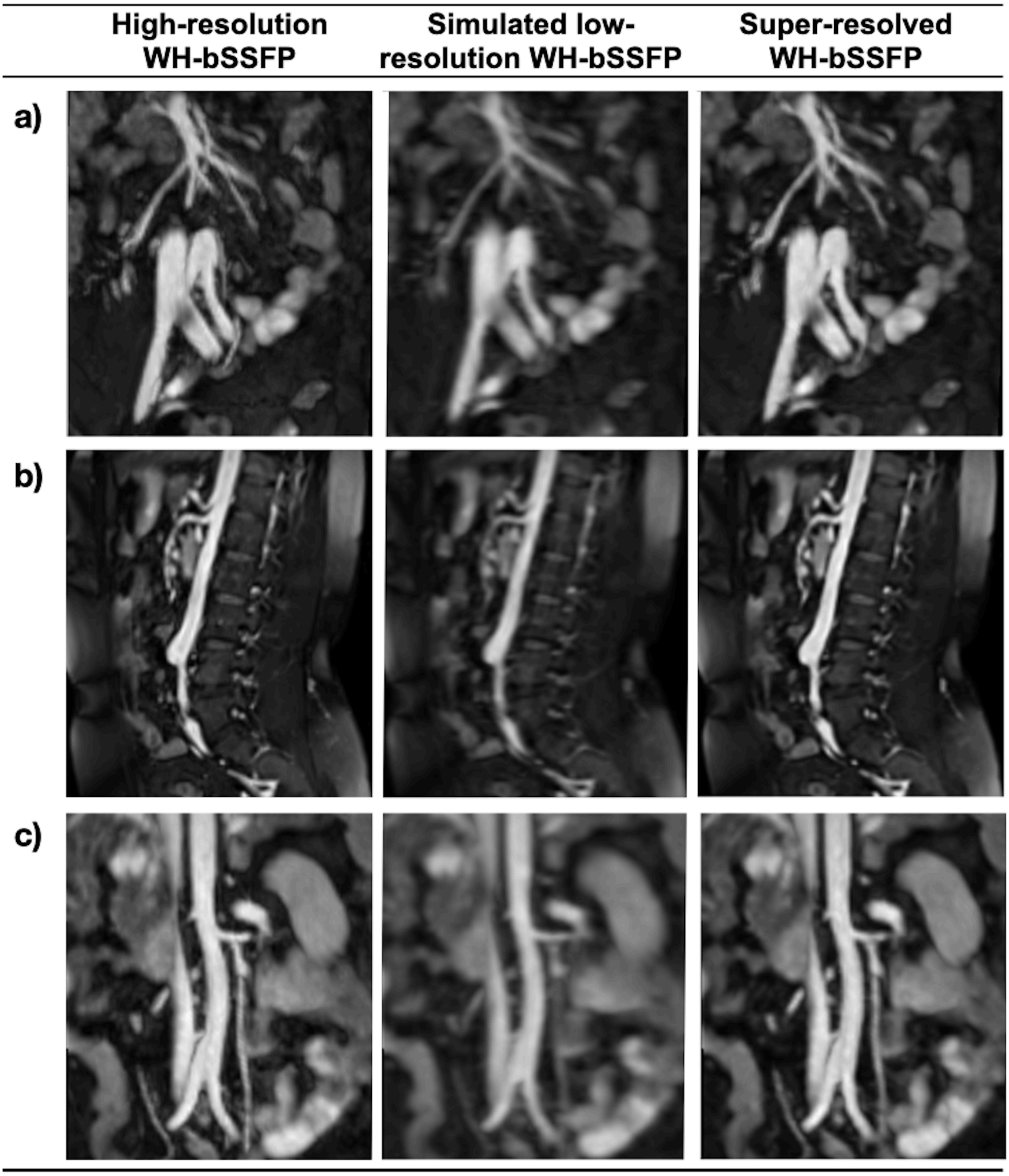

Left: Original high-resolution WH-bSSFP abdominal data, Middle: Simulated low-resolution WH-bSSFP abdominal data, Right: Resulting super-resolved abdominal data. Multiplanar reformats of the abdominal vasculature in a volunteer. a) shows the confluence of the Iliac veins, bifurcation of the abdominal aorta and the mesenteric vessels, b) shows the abdominal aorta, coeliac artery and superior mesenteric artery, and c) shows the abdominal aorta, left renal artery and left gonadal artery. Note the improved visualisation of small vessels in the super-resolution reconstruction compared to the low resolution images.
